# Posttranslationally modified self-peptides promote hypertension in mouse models

**DOI:** 10.1172/JCI174374

**Published:** 2024-08-15

**Authors:** Nathaniel Bloodworth, Wei Chen, Kuniko Hunter, David Patrick, Amy Palubinsky, Elizabeth Phillips, Daniel Roeth, Markus Kalkum, Simon Mallal, Sean Davies, Mingfang Ao, Rocco Moretti, Jens Meiler, David G. Harrison

**Affiliations:** 1 Division of Clinical Pharmacology, Department of Medicine,; 2 Department of Medicine, and; 3 Division of Infectious Diseases, Department of Medicine, Vanderbilt University Medical Center, Nashville, Tennessee, USA.; 4 Institute for Immunology and Infectious Diseases, Murdoch University, Murdoch, Australia.; 5 Center for Drug Safety and Immunology, Vanderbilt University Medical Center, Nashville, Tennessee, USA.; 6 Department of Immunology and Theranostics, Beckman Research Institute, City of Hope, Duarte, California, USA.; 7 Center for Structural Biology, and; 8 Department of Chemistry, Vanderbilt University, Nashville, Tennessee, USA.; 9 Institute for Drug Discovery, Universität Leipzig Medical School, Leipzig, Germany.

**Keywords:** Cardiology, Immunology, Antigen, Hypertension, MHC class 1

## Abstract

Posttranslational modifications can enhance immunogenicity of self-proteins. In several conditions, including hypertension, systemic lupus erythematosus, and heart failure, isolevuglandins (IsoLGs) are formed by lipid peroxidation and covalently bond with protein lysine residues. Here, we show that the murine class I major histocompatibility complex (MHC-I) variant H-2D^b^ uniquely presents isoLG-modified peptides and developed a computational pipeline that identifies structural features for MHC-I accommodation of such peptides. We identified isoLG-adducted peptides from renal proteins, including sodium glucose transporter 2, cadherin 16, Kelch domain–containing protein 7A, and solute carrier family 23, that are recognized by CD8^+^ T cells in tissues of hypertensive mice, induce T cell proliferation in vitro, and prime hypertension after adoptive transfer. Finally, we find patterns of isoLG-adducted antigen restriction in class I human leukocyte antigens that are similar to those in murine analogs. Thus, we have used a combined computational and experimental approach to define likely antigenic peptides in hypertension.

## Abstract 2

Specific self-peptides undergo posttranslational modification to induce hypertension and mediate inflammation.

## Graphical abstract

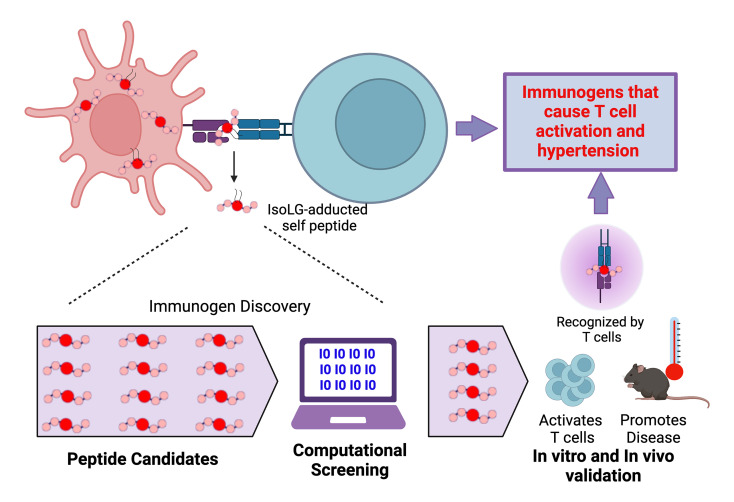

## Introduction

Accumulating evidence from the last several decades implicates inflammation and immune activation in the genesis of hypertension and its sequelae (1). Both the innate and adaptive immune systems contribute to hypertension-associated inflammation (2), but T cell–mediated responses in particular play an especially critical role. In 2007, Guzik et al. demonstrated that mice deficient in mature T and B cells (*Rag1^–/–^* mice) were protected from both angiotensin II– and deoxycorticosterone acetate and sodium chloride–induced (DOCA salt–induced) hypertension. Adoptive transfer of T cells, but not B cells, restored the hemodynamic phenotype (3). Deletion of CD247 (CD3 ζ chain), which selectively eliminates T cells, reduced both hypertension and renal inflammation and kidney dysfunction in Dahl salt–sensitive rats (4). Angiotensin II also induced T cell accumulation in the aortas and kidneys of mice with a humanized immune system, and increased levels of both effector memory CD4^+^ and CD8^+^ T cells that produce IFN-γ and IL-17A in hypertensive humans. Likewise, CD8^+^ T cells with a senescent phenotype have been observed in the peripheral blood of hypertensive humans compared with normotensive control individuals (5, 6).

While both CD8^+^ and CD4^+^ T cells contribute to the hypertensive phenotype (7, 8), selective depletion of CD8^+^ T cells in mice confers partial protection against hypertension, while CD4^+^ T cell depletion does not (9). Single-cell sequencing performed in this same study showed that an oligoclonal population of CD8^+^ T cells, but not CD4^+^ T cells, accumulates in the kidney but not in other tissues (9). These data imply the existence of antigens recognized by specific CD8^+^ T cells that are present in hypertension but not in normotensive conditions.

Posttranslational modification of self-proteins can produce novel antigens and is an important mechanism for activating immune responses in many diseases. In rheumatoid arthritis, citrullinated self-peptides activate helper T cell and humoral immune responses (10, 11). Covalent modification and noncovalent association of self-proteins and certain drugs or their metabolites (hapten formation) and can produce antigens that induce dramatic humoral and T cell responses in drug hypersensitivity (12, 13). Mass spectrometry data from human tumors also confirm a variety of posttranslationally modified cancer antigens, some of which activate tumor-specific T cell responses (14).

In hypertension, CD8^+^ T cell activation is closely linked to excessive production of reactive oxygen species (ROS), resulting in the formation of electrophiles, including isolevuglandins (IsoLGs). IsoLGs are produced by free radical–mediated oxidation of arachidonic acid (15). They are highly reactive intermediates that rapidly form covalent bonds with free amines, especially lysine residues in proteins (16–18). IsoLG-adducted proteins accumulate in multiple inflammatory and cardiovascular diseases closely related to hypertension, including systemic lupus erythematosus (19), atrial fibrillation (20), atherosclerosis (21), and nonischemic heart failure (22). There is a marked increase in IsoLG adducts in DCs of hypertensive compared with normotensive mice and scavenging of IsoLGs with 2-hydroxybenzylamine (2-HOBA) to inhibit adduct formation attenuates hypertension and end-organ damage in experimental hypertension. Adoptive transfer of DCs from hypertensive mice primes hypertension in recipient mice, and this is prevented if the donor mice have received 2-HOBA or if the recipient mice lack T cells. Likewise, adoptive transfer of DCs in which IsoLGs have been induced ex vivo primes hypertension in recipient mice. Further data in animal models show that T cells isolated from hypertensive mice proliferate when exposed to DCs presenting IsoLG-modified proteins (23). The percentage of monocytes containing IsoLGs is increased in hypertensive humans compared with normotensive individuals (23). Taken together, these data suggest that IsoLG-adducted peptides act as antigens for the activation of T cells in hypertension (24). We have also shown that factors common to the hypertensive milieu, including catecholamines (25), excess sodium (26, 27), and altered mechanical forces (28) increase formation of IsoLG adducts in antigen-presenting cells. Understanding the specific peptides that are IsoLG adducted in hypertension would be extremely informative, providing insight into the cells and subcellular locations where this pathologic process occurs and provide therapeutic opportunity to intervene and arrest it.

In the present work, we identify self-peptides that serve as substrates for IsoLG adduction and CD8^+^ T cell activation. By leveraging several publicly available and custom-developed computational tools and workflows, we were able to identify a limited library of candidates that we individually tested in vitro and in vivo. We show that several of these candidate IsoLG-adducted peptides are recognized by CD8^+^ T cells, induce CD8^+^ T cell activation, and promote hypertension in mice. To our knowledge, these studies are the first to define specific self-peptides that, when adducted by IsoLG, are responsible for T cell–mediated inflammation in hypertension, carrying significant implications for the future treatment of hypertension and related illnesses.

## Results

### IsoLG-adducted peptides are H-2D^b^ restricted.

**Figure 1:**
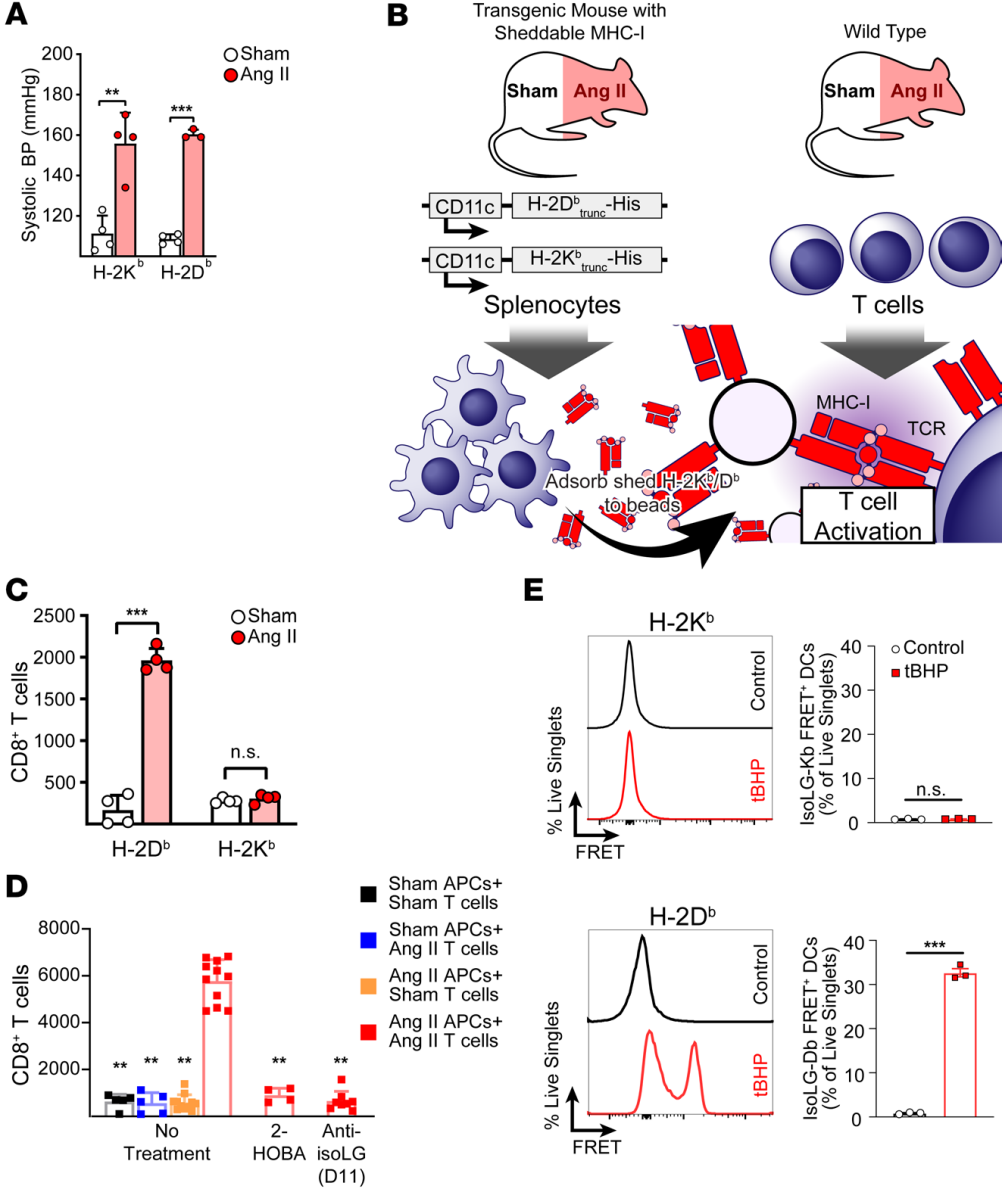
Presentation of IsoLG-adducted peptides is H-2D^b^ restricted. (**A**) Transgenic mice develop hypertension in response to angiotensin II (Ang II) (*n* = 4). **P < 0.01, ***P < 0.001 by Student’s *t* test. (**B**) Transgenic mice expressing soluble forms of H-2D^b^ or H-2K^b^ were treated with Ang II to induce hypertension before splenocyte harvesting and culture. Shed MHC-I was adsorbed onto Ni-agarose beads, cocultured with T cells from WT mice, and T cell proliferation measured with serial dye dilution and flow cytometry. (**C**) CD8^+^ T cells proliferate if exposed to bead-bound H-2D^b^, but not H-2K^b^ (*n* = 4). ***P < 0.001 by Student’s *t* test. (**D**) CD8^+^ T cell proliferation is only observed if both soluble H-2D^b^ and T cells are isolated from Ang II–treated animals, and treating transgenic mice with the IsoLG scavenger 2-HOBA or blocking T cell–MHC-I interactions with the anti-IsoLG antibody D11 inhibits CD8^+^ T cell activation (*n* = 4–11). APCs, antigen-presenting cells. **P < 0.01 vs. No Treatment Ang II APCs + Ang II T cells by 2-way ANOVA and Holm-Šidák post hoc test. (**E**) After treating mouse DCs with tBHP to induce IsoLG adduct formation, cells were stained with antibodies for MHC-I and IsoLG conjugated to a complementary FRET fluorophore pair. FRET signal was observed when staining for H-2D^b^ and IsoLG in tBHP-treated DCs, but not untreated cells or when staining for H-2K^b^ (*n* = 3). All data are presented as mean ± SD. ***P < 0.001 by Student’s *t* test.

The class I major histocompatibility complex (MHC-I) displays selective peptide repertoires dictated by the amino acid composition of the antigen binding cleft (29) and can display antigens with posttranslational modifications that generate an autoreactive CD8^+^ T cell response in a variety of diseases (30–35). We hypothesized that IsoLG-adducted peptides are similarly restricted in MHC-I presentation and that this restriction modulates T cell recognition. To test this, we generated 2 transgenic mouse strains expressing respectively truncated forms of 1 of 2 major MHC-I alleles found in C57BL/6 mice: H-2K^b^ or H-2D^b^. Each transgene was driven by a CD11c promoter and possessed both a His tag and a truncated transmembrane domain, allowing for extracellular secretion of MHC-I and its bound peptide (Supplemental Table 1 and Supplemental Figure 1; supplemental material available online with this article; https://doi.org/10.1172/JCI174374DS1). Transgenic animals were treated for 2 weeks with angiotensin II or a sham infusion. In both strains, angiotensin II induced a similar degree of hypertension (Figure 1A). This was expected because nothing was done to disrupt the endogenous MHC-I in these mice. Splenocytes from these mice were then placed in culture for 3 days and the shed MHC-I adsorbed onto Ni-agarose beads (Figure 1B). The MHC-I–loaded beads were then used to stimulate splenic CD8^+^ T cells from either hypertensive or sham-infused WT mice that had been preloaded with the CellTrace CFSE proliferation marker. T cell proliferation was measured by assessing dye dilution. We found that T cells exposed to bead-bound and antigen-loaded H-2D^b^ proliferated, while those exposed to H-2K^b^ did not (Figure 1C). Furthermore, we observed T cell proliferation only when both T cells and bead-bound H-2D^b^ were isolated from hypertensive animals and not mice that had received sham infusion (Figure 1D). Treating the donor transgenic animals with the IsoLG scavenger 2-HOBA or adding to the culture a single-chain variable fragment antibody that binds all IsoLG adducts (D11) prevented T cell proliferation, suggesting that T cell activation occurred in response to hypertension-specific IsoLG-adducted antigens.

To determine whether IsoLG-adducted antigen restriction was due to relative differences in MHC-I binding affinity for the modified peptide between H-2D^b^ or H-2K^b^ or differences in T cell receptor (TCR) recognition of the MHC-modified peptide complex, we treated murine DCs with *tert*-butyl hydroperoxide (tBHP), which we have shown stimulates IsoLG formation, and stained with antibodies specific for either H-2D^b^ or H-2K^b^ and IsoLG-adducted peptides. Antibodies were conjugated to complementary FRET fluorophore pairs, with a positive FRET signal indicating proximity between IsoLG-adducted peptides and either H-2D^b^ or H-2K^b^. tBHP-treated DCs stained with anti–H-2D^b^ generated a positive FRET signal, while untreated cells or tBHP-treated DCs stained with H-2K^b^ did not (Figure 1E). These results indicate that H-2D^b^ can present IsoLG-adducted peptides, promoting T cell activation, while H-2K^b^ cannot.

### Computational screening identifies peptide residue positions favoring IsoLG adduction.

Peptide binding affinity for MHC-I is largely dictated by structural constraints imposed by the MHC-I peptide binding cleft that, unlike the groove of MHC-II, is closed and accommodates shorter peptides 8–10 amino acids long (36–38). We reasoned that the IsoLG-adducted lysine imposed significant limitations on a peptide’s ability to take on certain structural conformations, and that understanding these limitations might help narrow the list of possible peptides serving as substrates for IsoLG adduction. To test this hypothesis, we developed a computational pipeline for modeling IsoLG-adducted peptides bound to MHC-I using FlexPepDock refinement. FlexPepDock refinement is a protocol implemented in the protein modeling software suite Rosetta, developed to model receptor-bound peptides and used previously to predict MHC-I–peptide structures and peptide-receptor binding affinity with a high degree of accuracy (39–41).

**Figure 2:**
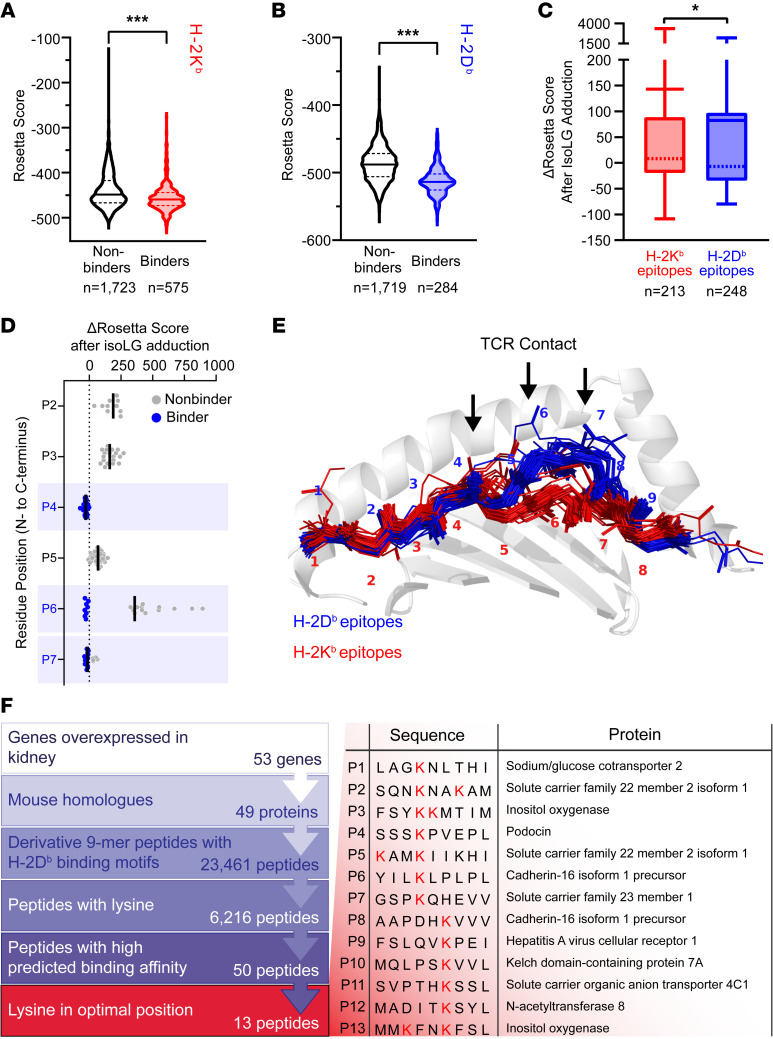
A computational modeling pipeline predicts IsoLG-adducted peptides presented by H-2D^b^. (**A** and **B**) Rosetta scores are more favorable (more negative) for peptides known to bind to H-2K^b^ (**A**) and H-2D^b^ (**B**) when compared with known nonbinders. ***P < 0.001 by Mann-Whitney test. (**C**) When lysine-containing peptides are adducted with IsoLG in silico, Rosetta predicts smaller (more favorable) score changes if those peptides are bound to H-2D^b^ compared with H-2K^b^ (solid bars = mean, dashed line = median, box boundaries = interquartile range, error bars ± min/max values). *P < 0.05 by Mann-Whitney test. (**D**) Rosetta score changes after in silico IsoLG adduction for all nonanchoring residues in lysine-containing H-2D^b^–bound epitopes predict residue sites 4, 6, and 7 as energetically favorable positions (positions at which multiple peptides modeled have no score increase after adduction. Black bars indicate mean Δ Rosetta score). (**E**) These residues correspond to areas recognized by T cell receptors interacting with H-2D^b^ epitopes. (**F**) Strategy for identifying a library of peptide candidates to screen in vitro and in vivo.

Using preexisting structural templates, we benchmarked FlexPepDock refinement on all peptide–MHC-I structures available in the protein data bank, generating high-fidelity models (Supplemental Figure 2, A–C, and Supplemental Table 2). We next generated structures for H-2D^b^– and H-2K^b^–bound epitopes with known binding affinity available in the Immune Epitope Database (IEDB) and compared the Rosetta energy score terms for known binders (≤500 nm IC_50_) and nonbinders (>500 nm IC_50_) (42). For both H-2D^b^ and H-2K^b^ epitopes, known MHC-I binders had on average lower, or more favorable, Rosetta energies than nonbinders (Figure 2, A and B). We then selected known binders to H-2D^b^ or H-2K^b^ containing at least 1 lysine residue and measured changes in Rosetta energy after in silico IsoLG adduction. H-2K^b^–bound epitopes displayed a greater increase in Rosetta energy after IsoLG adduction than H-2D^b^–bound epitopes (Figure 2C), suggesting an inability to accommodate this posttranslational modification.

We next examined per-residue energy changes for H-2D^b^–bound epitopes 8 or 9 residues in length before and after IsoLG adduction for all non-anchoring residues and found that peptides with lysine at residue positions 4, 6, or 7 showed the smallest changes in Rosetta energy following IsoLG adduction (Figure 2D). These positions correspond to solvent-accessible sites that are largely responsible for dictating T cell recognition in H-2D^b^–restricted epitopes (Figure 2E) (43).

Leveraging this insight, we produced a limited library of peptide candidates for further screening, each containing lysine at one of the optimal IsoLG adduction residue positions. Given our prior evidence that the kidney is a likely source of IsoLG-adducted peptides in hypertension, we focused our initial search on proteins with relative overexpression in the kidney (44). Of the 53 candidates identified, 49 had corresponding mouse homologs. We next identified peptide sequences derived from those proteins with H-2D^b^ binding motifs, predicted by the webtool NetMHCpan 4.0 to bind to H-2D^b^ with high affinity (“strong binders” are defined by a percentage rank score unique to NetMHCpan) (45). This approach yielded 13 peptides with at least 1 lysine in a position favoring IsoLG adduction as predicted by Rosetta. These sequences and the proteins from which they are derived are summarized in Figure 2F and Supplemental Table 3.

### IsoLG-adducted candidate peptides stimulate T cell proliferation in vitro and are recognized by T cells in end-organ tissues of hypertensive mice.

**Figure 3:**
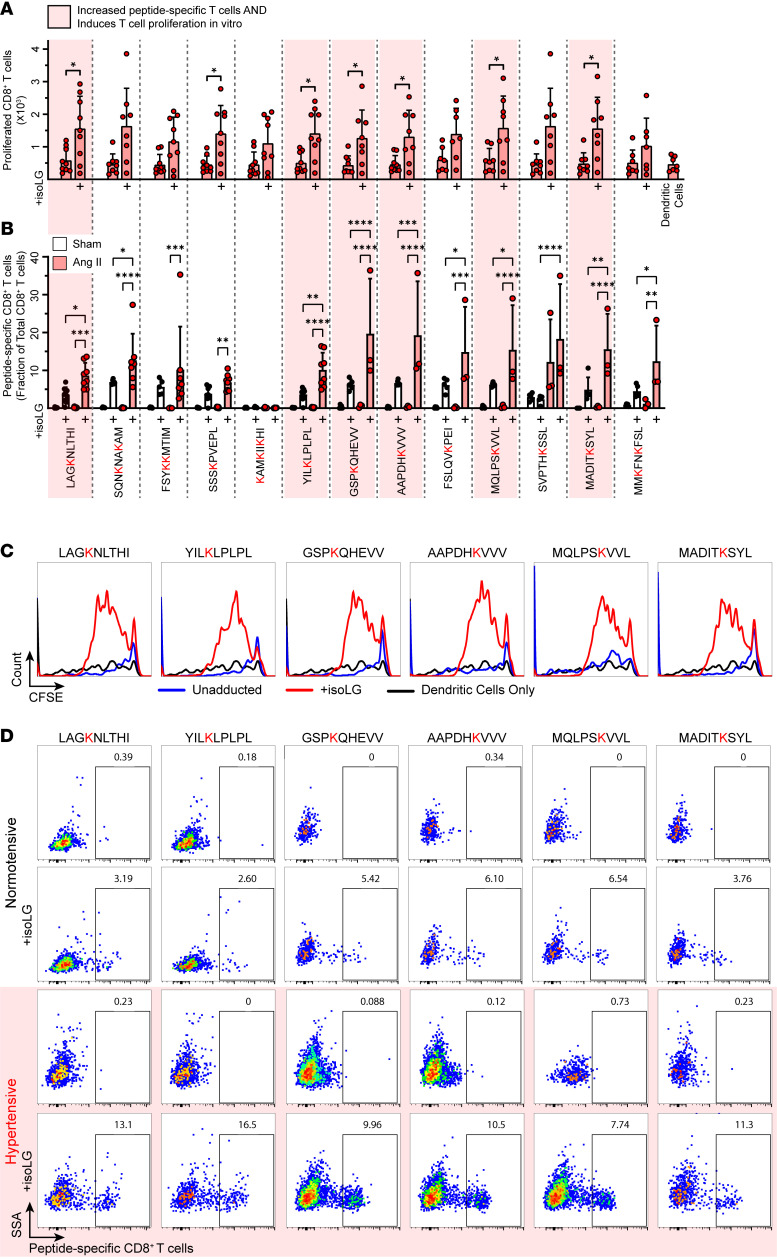
A subset of candidate IsoLG-adducted peptides are recognized by CD8^+^ T cells enriched in the aortas of hypertensive mice and induce CD8^+^ T cell proliferation in vitro. (**A**) Seven candidate IsoLG-adducted peptides induce the proliferation of T cells isolated from the bone marrow of hypertensive mice, while unadducted peptides do not (*n* = 7–9, mean ± SD). *P < 0.05 by Student’s *t* test. (**B**) Nine IsoLG-adducted peptides identify a population of peptide-specific CD8^+^ T cells that are enriched in the aortas of hypertensive mice (*n* = 3–9, mean ± SD). *P < 0.05, **P < 0.01, ***P < 0.001, ****P < 0.0001 by 2-way ANOVA and Holm-Šidák post hoc test. Six candidates are both recognized by CD8^+^ T cells and induce CD8^+^ T cell proliferation in vitro (highlighted in red). (**C**) Representative histograms illustrating proliferation of CD8^+^ T cells after exposure to each of these 6 candidate IsoLG-adducted peptides and (**D**) flow plots illustrating the increase in peptide-specific CD8^+^ T cells in the aorta.

**Figure 4:**
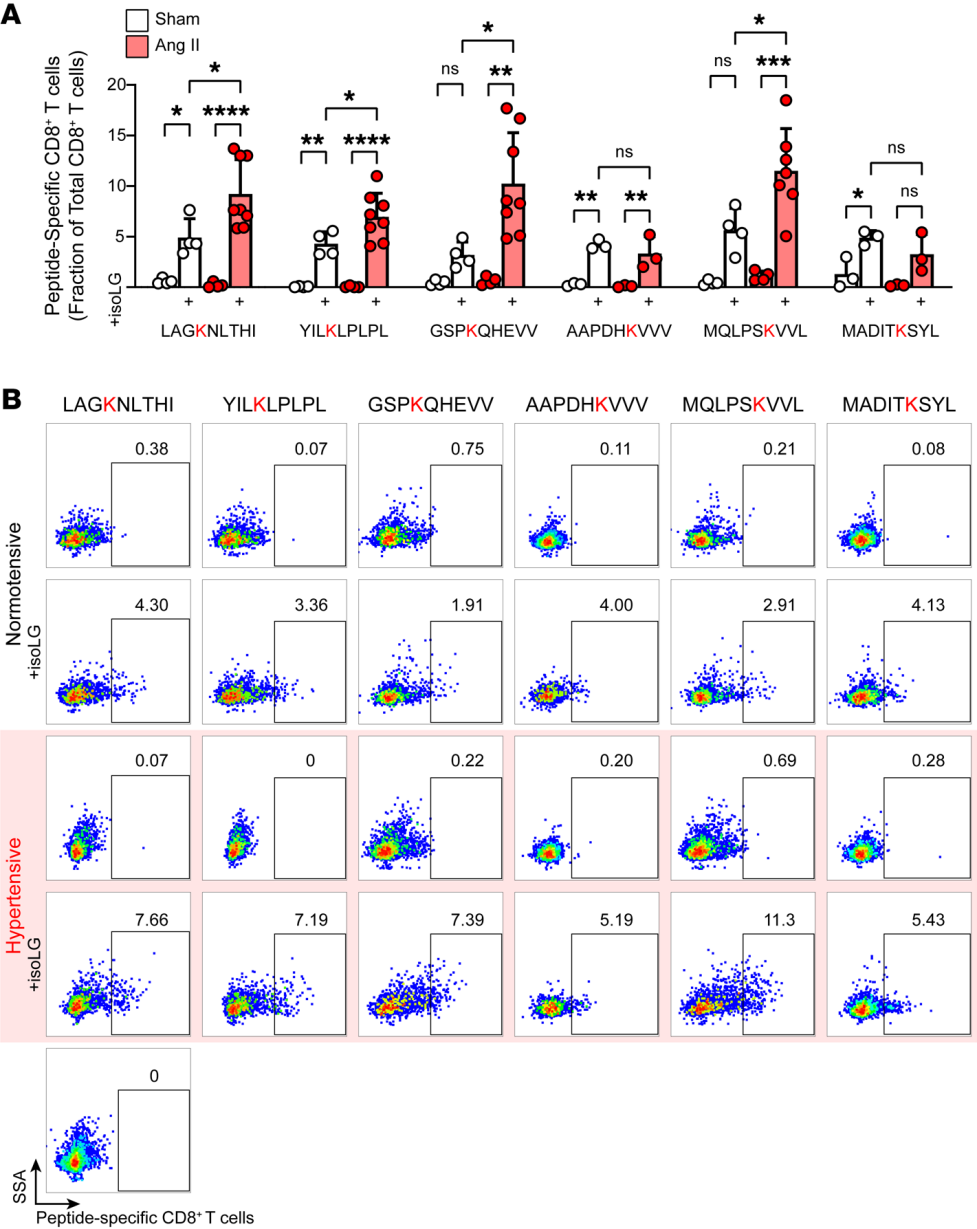
IsoLG-adducted peptide–specific CD8^+^ T cells are enriched in the kidneys of hypertensive animals. (**A**) Candidate peptides adducted with IsoLG are recognized by CD8^+^ T cells in the kidney and enriched after hypertension is induced with Ang II infusion (*n* = 3–8, mean ± SD). *P < 0.05; **P < 0.01; ***P < 0.001; ****P < 0.0001 by 2-way ANOVA and Holm-Šidák post hoc test. (**B**) Representative flow plots illustrating the increase in peptide-specific CD8^+^ T cells in the kidneys during hypertension.

**Figure 5:**
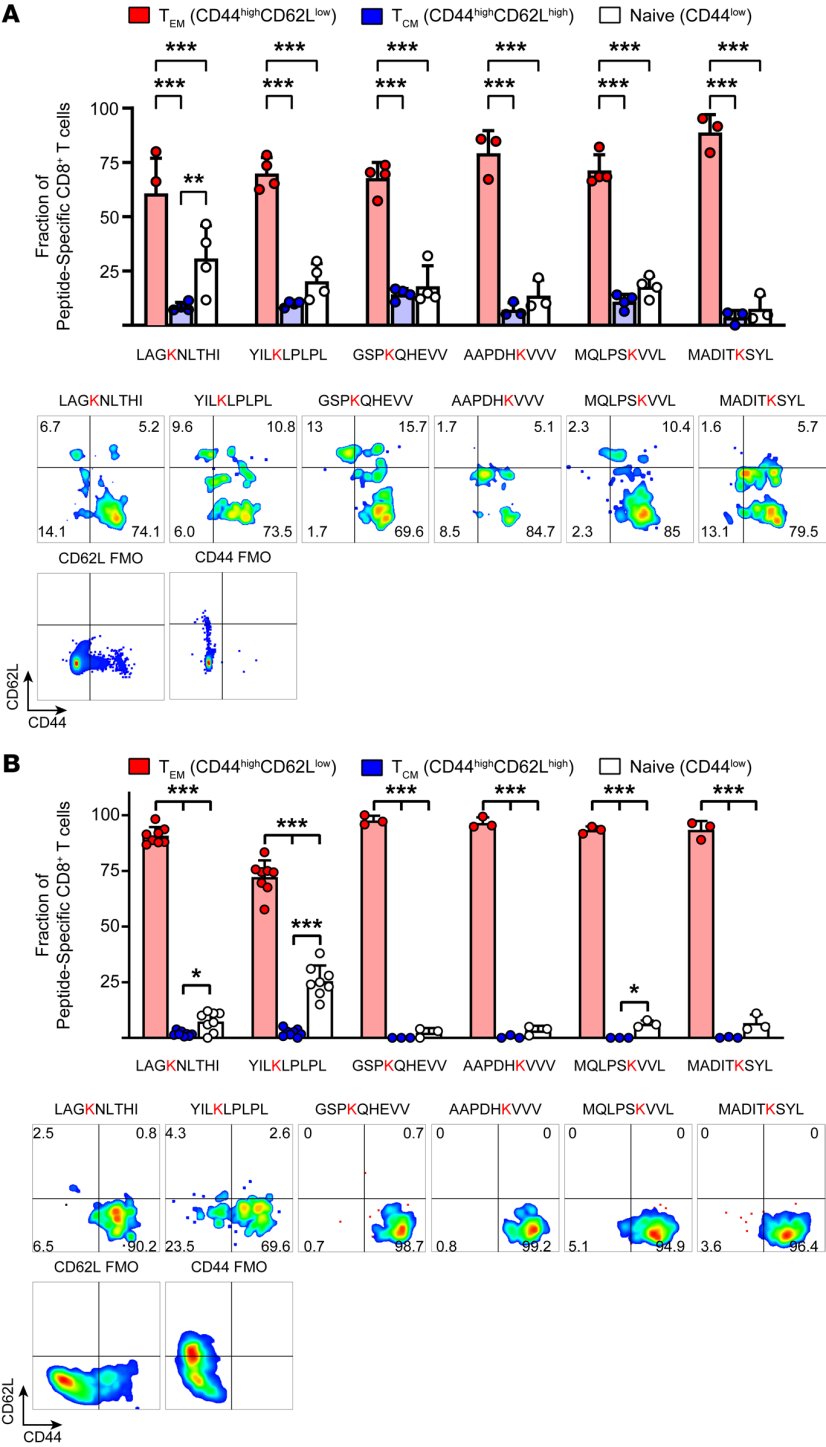
CD8^+^ T cells recognizing IsoLG-adducted peptides are predominantly memory T cells in the aorta and kidney. Staining for memory T cell markers CD44 (all memory cells) and CD62L (central memory cells) reveals that IsoLG-adducted peptide–specific CD8^+^ T cells are primarily effector memory cells in the kidney (**A**) and in the aorta (**B**) (*n* = 3–8, mean ± SD). FMO, fluorescence-minus-one control. *P < 0.05; **P < 0.01; ***P < 0.001 by 1-way ANOVA and Holm-Šidák post hoc test.

We isolated T cells from the bone marrow of angiotensin II–treated mice and exposed them to DCs pulsed with each candidate peptide, measuring T cell proliferation by serial dye dilution. Seven of 13 candidate peptides induced a statistically significant increase in proliferation of CD8^+^ T cells from hypertensive, but not normotensive, mice (Figure 3A and Supplemental Figure 3). To test T cell specificity for each candidate peptide, we employed a fluorescently tagged H-2D^b^–IgG1 fusion protein loaded with each candidate, IsoLG adducted or not. Of the 13 peptides screened, 9 were recognized by CD8^+^ T cells from the aortas of hypertensive mice to a greater extent than observed in mice without hypertension (Figure 3B and Supplemental Figure 4). This analysis revealed that up to 14% of CD8^+^ T cells in aortas of hypertensive mice recognized individual candidate peptides that were IsoLG adducted. T cells did not recognize peptides unadducted by IsoLGs. Six candidates induced proliferation in T cells from angiotensin II–treated mice and identified CD8^+^ T cells enriched in the aortas in hypertension (Figure 3, C and D). These same 6 IsoLG-adducted peptides also induced proliferation of CD4^+^ T cells in vitro, suggesting that they are also likely capable of being presented by class II MHC and can activate a broader immune response (Supplemental Figure 5). We also performed flow cytometry on kidney homogenates, staining for CD8^+^ T cells specific for the 6 candidate IsoLG-adducted peptides that are recognized by aortic T cells and capable of inducing T cell proliferation. We confirmed that CD8^+^ T cells recognizing 4 of the 6 IsoLG-adducted peptides enriched in the aortas of hypertensive mice were also increased in the kidneys compared with normotensive controls (Figure 4, A and B). Staining for memory T cell markers CD44 and CD62L revealed that CD8^+^ T cells specific for these IsoLG-adducted peptides are predominantly effector memory cells in both the kidney (Figure 5A) and in the aorta (Figure 5B) of hypertensive animals. This same trend is evident for IsoLG-adducted peptide–specific CD8^+^ T cells isolated from the aortas and kidneys of normotensive controls (Supplemental Figure 6).

Given that the bone marrow serves as a reservoir for hypertension-specific CD8^+^ memory T cells (25, 46), we also performed flow cytometry on single-cell suspensions from the bone marrow of angiotensin II–treated mice. We only observed increases in the fraction of CD8^+^ T cells recognizing our IsoLG-adducted peptides for 2 of the 6 peptides of interest (Supplemental Figure 7A). For all 6 peptides of interest, the peptide-specific CD8^+^ T cells were predominantly a mixture of effector and memory cells in the bone marrow (Supplemental Figure 7, B and C).

### IsoLG-modified candidate peptides augment hypertension in vivo.

**Figure 6:**
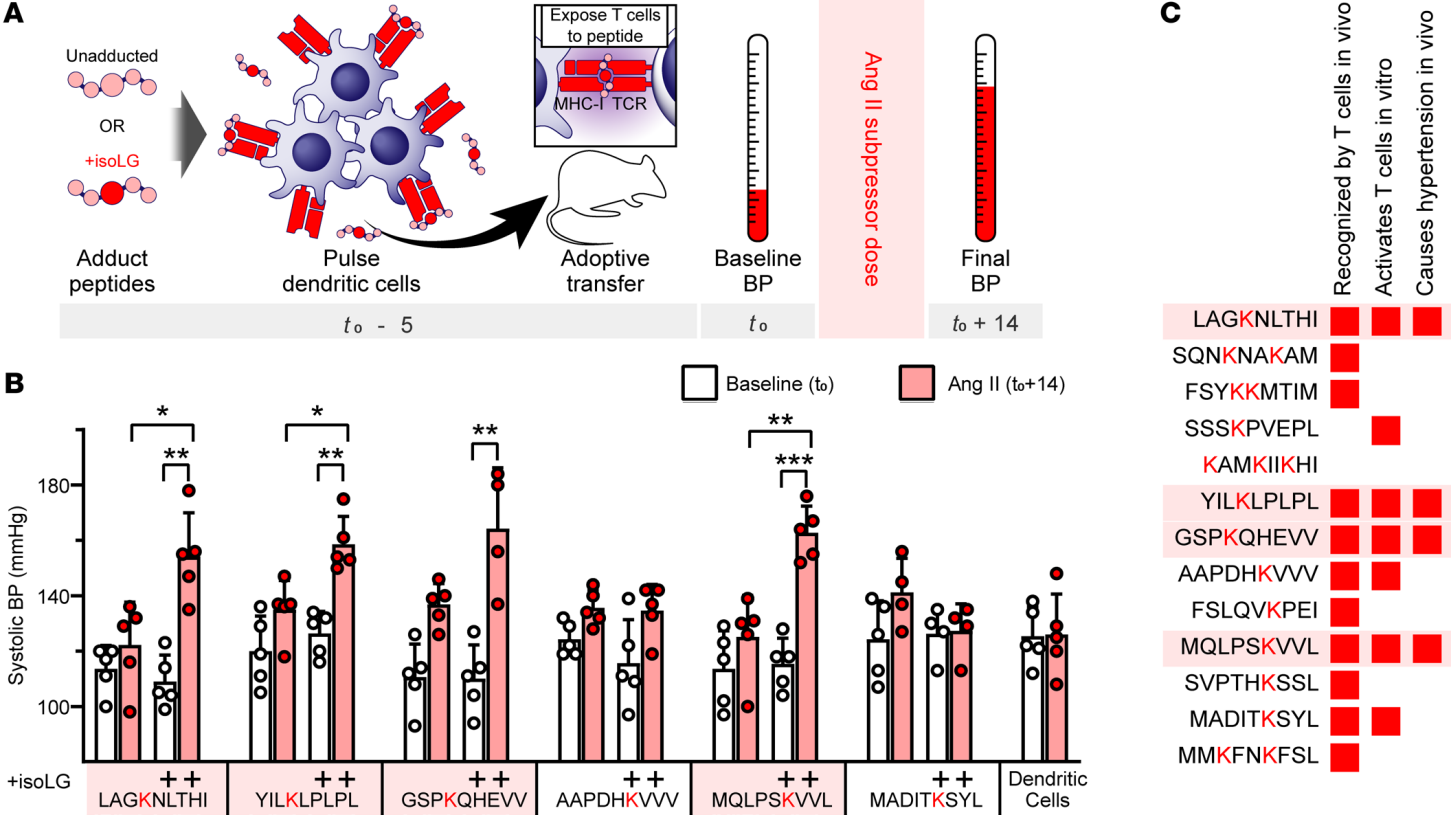
IsoLG-adducted peptides induce hypertension in mice. (**A**) Experimental diagram. IsoLG-adducted peptide candidates and their unadducted counterparts were loaded onto DCs and adoptively transferred prior to 14 days of treatment with a subpressor dose of Ang II. (**B**) Four of the 6 candidates tested induced a significant increase in blood pressure following adoptive transfer, while unadducted peptides did not (*n* = 4–5, mean ± SD). *P < 0.05; **P < 0.01; ***P < 0.001 by 2-way ANOVA and Holm-Šidák post hoc test. (**C**) Of the original 13 candidates screened, 4 are recognized by CD8^+^ T cells, induce CD8^+^ T cell activation, and induce hypertension in mice following adoptive transfer.

To test the potential roles of each candidate peptide in hypertension, we performed the experiment illustrated in Figure 6A. Briefly, CD11c^+^ DCs were pulsed overnight with candidate peptides with or without IsoLG adduction. These DCs were then adoptively transferred to WT mice and 5 days later an infusion of a generally subpressor dose of angiotensin II was begun (23). Blood pressures were measured before and after 2 weeks of angiotensin II treatment. Of the 6 peptides tested (those that labeled CD8^+^ T cells in the aorta and induced CD8^+^ T cell proliferation in vitro), 4 induced blood pressure elevations as high as 180 mmHg after adoptive transfer (Figure 6B). Flow cytometric profiling of the CD8^+^ T cells in the aortas and kidneys of these animals revealed statistically significant increases in the fraction of peptide-specific CD8^+^ T cells following adoptive transfer and treatment with low-dose angiotensin II. Treatment with low-dose angiotensin II alone, or adoptive transfer to IsoLG-adducted peptides alone, did not induce a similar increase (Supplemental Figure 8).

Figure 6C summarizes the stepwise screening of our candidate peptides. Of the 13 peptides originally identified by our computational analysis, 10 identified T cells enriched in the target tissues of hypertensive mice. Seven of these peptides induced proliferation of CD8^+^ T cells from hypertensive mice, and 4 augmented hypertension in vivo.

To ensure these findings were not specific to the angiotensin II–induced hypertension model, we also induced hypertension with the nitric oxide synthase inhibitor *N*_ω_-nitro-L-arginine methyl ester (L-NAME). Aortas were harvested and single-cell suspensions stained for CD8^+^ T cells recognizing each of the 4 IsoLG-adducted peptides found to augment hypertension in angiotensin II–treated animals. Animals treated with L-NAME had a significantly higher fraction of peptide-specific T cells in their aortas than normotensive animals, with up to 35% of CD8^+^ T cells recognizing IsoLG-adducted epitopes (Supplemental Figure 9).

To obtain evidence that the proteins from which our peptides of interest are derived are IsoLG adducted in vivo, we immunoprecipitated all IsoLG-adducted proteins from kidney homogenates isolated from normotensive and hypertensive mice. We then performed Western blotting using anti-SGLT2 (the protein from which the LAGKNLTHI peptide is derived) and anti-CDH16 (the YILKLPLPL peptide). The relative abundance of IsoLG-adducted SGLT2 was significantly increased in hypertensive mice, and there was a trend for an increase in IsoLG-adducted CDH16 in the kidneys of hypertensive mice compared with sham controls (Supplemental Figure 10).

We performed mass spectrographic analysis of one of our synthesized immunogenic peptides (LAGKNLTHI) following ex vivo IsoLG adduction. The overall signal strength for the adducted peptide was lower than that of the unadducted peptide (Supplemental Figure 11). Ion chromatograms demonstrated the presence of multiple diastereomers, including pyrrole, anhydrolactam, and anhydropyrrole products all present in the same sample (Supplemental Figure 12).

### Class I human leukocyte antigens exhibit differential presentation of IsoLG-adducted peptides.

**Figure 7:**
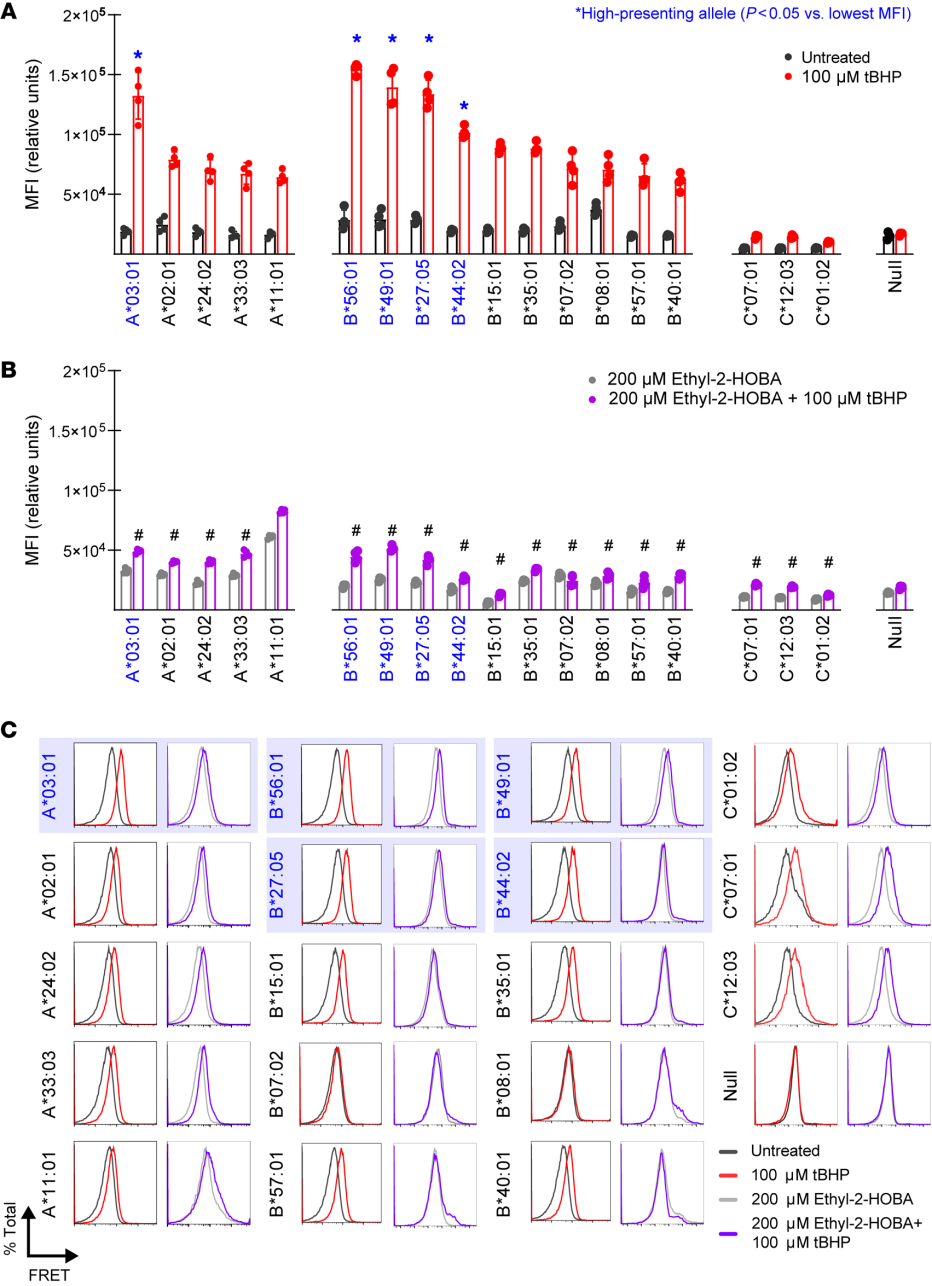
IsoLG-adducted peptides are preferentially displayed by certain HLA molecules. (**A**) Treating K562 cells expressing single HLA alleles with tBHP induces a significant increase in the HLA-IsoLG FRET proximity mean fluorescence intensity (MFI) for all alleles screened, excepting the HLA-null control. However, there are certain alleles with significantly higher FRET MFI compared with the lowest measured in each allele class (**A**–**C**) (*n* = 4, mean ± SD). *P < 0.05 vs. lowest average MFI for corresponding allele class by 2-way ANOVA and Holm-Šidák post hoc test. (**B**) Treatment with the dicarbonyl scavenger ethyl-2-HOBA significantly reduces FRET MFI compared with tBHP-only-treated cells (*n* = 4). ^#^*P* < 0.05 vs. corresponding tBHP-treated group by 2-way ANOVA with Holm-Šidák post hoc test. (**C**) Example FRET signal for all HLA alleles tested. “High-presenting” HLA-A and HLA-B alleles are highlighted in blue.

The above data from mice strongly suggest that T cell recognition of IsoLG-adducted peptides depends on their affinity for a given MHC-I. To determine whether IsoLG-adducted peptides are similarly restricted in their presentation among human MHC-I variants, we identified class I human leukocyte antigen (HLA) variants from a curated database of HLA allele frequencies (47). We selected HLA alleles for each of 3 subpopulations from the US National Merit Donor Program with a phenotype frequency of greater than 5%, leaving 18 HLA alleles in total after excluding duplicates between subpopulations (Supplemental Table 4). Cells lacking native HLA expression (HLA null) were transfected with each allele, treated with tBHP to induce IsoLG formation, and assayed for IsoLG adduct presentation as in Figure 1E using FRET and flow cytometry. We found significant variability of FRET signal between various HLA-A and HLA-B alleles, suggesting that certain alleles are more adept at displaying IsoLG-adducted peptides (Figure 7A). Scavenging IsoLGs with ethyl-2-HOBA significantly reduced FRET signal across all HLA alleles tested (Figure 7B).

We identified 1 HLA-A variant and 4 HLA-B variants with an enhanced FRET signal, suggesting these alleles can display IsoLG-adducted peptides (Figure 7C). Using FlexPepDock, we selected lysine-containing peptides, 9 residues in length and derived from proteins overexpressed in human renal tissue, predicted bind to the HLA in question with high affinity. We modeled the peptides to each HLA screened before and after IsoLG adduction. Rosetta energy scores were more favorable for IsoLG-adducted peptides docked to the HLA-A and HLA-B variants, with enhanced FRET signal (“high-presenters”) when compared with the remaining HLA variants (Supplemental Figure 13A). For the 3 high-presenting HLA variants with available crystal structures, we compared energy score changes at each residue position outside of the MHC-I binding pockets and for which there was at least 1 lysine-containing peptide. Similar to H-2D^b^–bound peptides, we found that favorable energy score changes after IsoLG adduction corresponded to more exposed sites with potentially greater TCR accessibility on representative HLA-bound peptides (Supplemental Figure 13B).

## Discussion

For decades, T cells have been identified in the peripheral tissues of humans with hypertension and in several experimental models. Evidence accumulated over the last 10 years strongly implies the existence of IsoLG-adducted peptides as antigens in hypertension (9, 23, 48). In the present work, we identify self-peptides that, when modified by IsoLG, are both recognized by and activate T cells in hypertensive mice and prime hypertension in vivo. These findings strongly suggest that the peptides identified in the current study or similar IsoLG-modified peptides play a role in the genesis of hypertension and its related end-organ damage.

The finding that T cells identified by these IsoLG-modified peptides are present in the aorta and kidney of hypertensive mice is compatible with prior work in which we showed that an oligoclonal population of CD8^+^ T cells accumulate in the kidneys of hypertensive mice and that T cells with a memory phenotype home to the bone marrow in hypertension (46). We have also shown that renal denervation prevents the appearance of activated DCs in secondary lymphoid organs and prevents the ultimate accumulation of T cells in the kidney (25). The observation of an increase in relative abundance of these cells in the aorta and kidney, but not the bone marrow, could also indicate demarginalization as they migrate to peripheral tissues. Taken together and with our current data, a paradigm emerges in which antigens in the kidney, like those identified in the current study, are presented to CD8^+^ T cells in secondary lymphoid organs. These activated T cells then home back to the kidney, vasculature, and the bone marrow, where they promote organ dysfunction and end-organ damage. We cannot exclude the possibility that these or similar antigens are formed in other organs and tissues.

In mouse models of obesity-associated insulin resistance, oligoclonal CD8^+^ T cell populations accumulate in response to IsoLG protein adducts presented by DCs located in adipose tissue (49). Oligoclonal CD8^+^ T cell populations are also found in human and murine atherosclerotic plaques, likely in response to plaque-specific antigens (21, 50, 51). In a murine model of heart failure, CD4^+^ T cells are activated after exposure to IsoLG-adducted cardiac peptides presented by class II MHC (22). We have also shown that IsoLG-adducted peptides contribute to systemic lupus erythematosus (19). It is unclear whether the peptides identified in the present study contribute to these other diseases; however, a combined computational and experimental approach like that employed here could be useful in these related conditions.

We found that CD8^+^ T cells recognizing IsoLG-adducted peptides in the aorta, kidney, and bone marrow are both effector and central memory cells. These findings are consistent with prior experiments performed in our laboratory illustrating that memory CD8^+^ T cells accumulate in the bone marrow and kidney in response to repeated hypertensive stimuli, and that memory T cell formation through CD70-mediated costimulation is necessary for hypertension pathogenesis (25, 46). Similar observations have been made in humanized mice, demonstrating increased numbers of memory T cells in the aorta and lymphatic tissues after induction of hypertension (5).

It is also of interest that even in nonhypertensive mice, 3% to 6% of CD8^+^ T cells in the aorta, kidney, and bone marrow recognize IsoLG-adducted peptides. In the peripheral tissues, these cells have the phenotype of effector memory cells. These findings suggest that these T cells may have previously encountered molecularly similar antigens. One such source of exposure could be antigens derived from commensal organisms or other chronic persistent foreign antigens that either mimic IsoLG adducts or that are themselves IsoLG adducted. Hypertension is closely associated with changes in gut microbiome content in humans (52). Germ-free mice are also protected against angiotensin II–induced hypertension and have reduced arterial and renal inflammatory infiltrate, and transplant of gut microbiome from hypertensive patients to germ-free animals can induce high blood pressure (53). Salt sensitivity, an important hypertension phenotype, is also intrinsically linked to both gut microbiome changes in mice and humans and is a potent stimulator for IsoLG production in DCs (54, 55). Whether or not the same T cells responsible for mediating inflammatory responses against gut bacteria (or some other viral antigen) also recognize IsoLG-adducted antigens is an important subject for further study.

The proteins we identified from which the IsoLG-adducted peptides are derived are all membrane-associated proteins and include transporters (in the case of LAGKNLTHI, derived from the sodium/glucose cotransporter encoded by *Slc5a2*, and GSPKQHEVV, derived from the sodium/vitamin C cotransporter encoded by *Slc23a1*), cell-cell adhesion proteins (YILKLPLPL, derived from the cell-cell adhesion protein encoded by *Cdh16*), and one without functional annotation (MQLPSKVVL, derived from the Kelch domain–containing protein encoded by *Klhdc7a*) (see Supplemental Table 3). LAGKNLTHI and GSPKQVEVV are derived from cytoplasmic regions of their respective proteins, while YILKLPLPL is derived from the extracellular region. These proteins may be adducted by IsoLG and processed via several pathways. Renal epithelial cells experience increased oxidative stress in hypertension (56, 57), and thus these proteins could undergo IsoLG adduction in their cell of origin. It is also possible that previously unadducted proteins, released with cellular debris or upon cell death in the kidney, are engulfed by DCs where they are then IsoLG adducted. In keeping with this, we have shown that superoxide production is increased by more than 6-fold in DCs of hypertensive mice and this is absent in mice lacking NOX2 (23).

IsoLGs are a family of 8 regioisomers, and the reaction of IsoLGs with lysine forms a number of adduct species, including pyrrole and oxidized pyrroles (58). Chromatograms derived from mass spectrometry data obtained from one of the immunogenic IsoLG-adducted peptides revealed characteristic signals congruent with different IsoLG adducts, including anhydrolactams and hydropyrroles (Supplemental Figure 12). While such differences in IsoLG structure might not appreciably change their affinity for MHC-I, different IsoLG regioisomer or adduct species could select for different T cell populations. This mechanism might explain why we observe many distinct T cell clonotypes in the kidneys of hypertensive mice, rather than a few single dominant clonal populations (9). Additionally, the robust T cell response observed despite a relatively low amount of IsoLG-adducted peptide (as assessed by mass spectrometry, Supplemental Figure 11) suggests that the adducts are high-quality immunogens.

Our data strongly suggest that IsoLG adducts are restricted to certain MHC-I variants in mice and preferentially displayed by certain HLA variants in humans. Such data imply there might exist a correlation between “high-risk” HLA variants and hypertension, as is the case for other diseases (59). There are several studies with small populations that correlate specific class II HLA alleles with hypertension severity, though this may be due to linkage disequilibrium rather than HLA itself (60, 61). A more recent study correlating phenotypes with imputed HLA alleles in a large clinical database did not reproduce these associations, and there is no single class I HLA that confers significantly elevated risk of hypertension (62). This may be due in part to the “mosaic” nature of hypertension, where distinct pathogenic stimuli act in concert to mediate a common phenotype (63). While single HLA alleles may be insufficient to promote disease, it is also possible that combinations of multiple alleles, or haplotypes, may confer either risk (if all are predisposed to IsoLG-adduct presentation) or protection (if they have low affinity for IsoLG adducts). The computational modeling pipeline employed for this study could also be employed to screen large numbers of antigen/HLA combinations. Further studies combining this screening tool with large databases containing both imputed HLA alleles and diagnosis codes may help identify high-risk haplotypes, and individual alleles later screened for IsoLG-adduct affinity in vitro.

There are several important limitations to consider for this work. Firstly, the peptides we screened are N-terminally acetylated. While necessary to prevent IsoLG adduction at the peptide N-terminus, N-terminal acetylation can also alter peptide binding affinity for MHC-I, resulting in false negatives in our screening studies (64). Solid-phase peptide synthesis using preadducted lysine residues could circumvent this problem, but an optimized protocol for manufacturing such residues is not yet available. In our initial computational screen, we also excluded peptides predicted to have a low binding affinity for H-2D^b^. This exclusion step reduced the number of peptides included in the screen by several orders of magnitude, but also may have rejected peptides with increased binding affinity for MHC-I following IsoLG adduction.

While we successfully identify the presence of self-proteins in IsoLG-adduct-enriched samples from which our peptides are derived, methods enabling the direct detection of the IsoLG-modified peptides themselves in vivo are lacking. Mass spectrometry can be routinely used to detect peptides eluted from MHC-I and MHC-II in cell culture, but routine data processing algorithms are limited in their ability to confidently identify peptide sequences with posttranslational modifications such as IsoLG, especially in the absence of prior reference data or biochemical enrichment (65). Developing these highly specialized tools to identify IsoLG-modified peptides in humans with hypertension is an active priority of our group and others. Furthermore, the use of mass spectrometry to detect protein and peptide fragments depends largely on the frequency of the chemical species of interest. As antigen potency is a property largely uncoupled from its frequency, mass spectrometry is limited in its ability to identify uncommon antigens and is unable to distinguish immunogens from benign peptides (66, 67). However, our computational pipeline circumvents these challenges and aids this discovery of IsoLG-modified peptides that independently illicit immune responses in vivo and in vitro.

Our data showed a robust proliferative response of CD4^+^ T cells after exposure to IsoLG-adducted peptides (Supplemental Figure 5). IL-17A–producing CD4^+^ T cells and γδ T cells are an important source of inflammation in hypertension and recognize IsoLG-adducted peptides in mouse models of nonischemic heart failure (8, 22, 68). Future studies should examine the ability of these cells to recognize and respond to similar IsoLG-adducted peptides and assess their presence and phenotype in target tissues affected by hypertension.

Despite these limitations, identifying antigens that mediate T cell–induced inflammation in hypertension is an important discovery with broad implications. Future work expanding the scope of immunogenic IsoLG-adducted self-peptides, identifying the T cells that recognize them, and characterizing molecularly similar antigens from human pathogens that may evoke cross-reactive memory T cell responses will enhance our understanding of how inflammation drives morbidity and mortality in this increasingly common ailment.

## Methods

### Sex as a biological variable.

Male mice were used exclusively for all animal experiments in this study. Male and female animals display differences in blood pressure and organ dysfunction associated with hypertension across multiple animal models, with male mice tending to develop more robust responses to various hypertensive stimuli (69, 70).

### Transgenic animals and murine hypertension models.

DNA fragments containing the H-2K^b^ and H-2D^b^ heavy chains with 6-His tags and without transmembrane domain regions downstream of a CD11c promoter were amplified and cloned into the pcDNA3.1 (H-2D^b^) and pET-3a (H-2K^b^) vectors. Resultant vectors were transformed into ampicillin-treated *E*. *coli* (DH5α), purified using the PureLink Maxiprep kit (Thermo Fisher Scientific), and sequenced to confirm the insertion. The resultant constructs were used to perform pronuclear injections for creation of transgenic C57BL/6 mice with soluble forms of H2-D^b^ and H2-K^b^, respectively.

To induce hypertension, male C57BL/6 mice (The Jackson Laboratory) were implanted with osmotic minipumps (Alzet) containing angiotensin II delivered at a rate of 490 ng/min/kg (regular dosing) or 140 ng/min/kg (subpressor dosing), or sodium acetate buffer (sham controls). Mice were studied at 12 weeks of age and received angiotensin II for 2 weeks, and blood pressure tail cuff measurements were made using the MC4000 Multichannel System for mice (Hatteras, Inc). The nitric oxide synthase inhibitor L-NAME (Sigma-Aldrich, N5751) was also used to induce hypertension. Twelve-week-old C57BL/6 mice were given L-NAME in drinking water (0.5 mg/mL) for 2 weeks. Control mice received facility drinking water. In some experiments, 2-HOBA was provided concurrently in drinking water at a concentration of 1 g/L. Animals were euthanized by CO_2_ asphyxiation prior to tissue isolation.

### In silico studies and computational screening.

Rosetta stable release version 3.13 (https://github.com/RosettaCommons/rosetta) was used for all computational studies with FlexPepDock refinement. We created Rosetta params files for modeling IsoLG-adducted lysine as previously described (71). Preliminary structures were generated by identifying the best-scoring preexisting template structure available in the PDB (after alignment and scoring with the Blocks Substitution Matrix BLOSUM62) and mutating peptide residues to match those of the query sequencing using the Rosetta Scripts mover MutateResidue prior to prepacking (72). MHC-I models were generated using AlphaFold2 (73). Unless otherwise specified, 250 models were generated for each peptide–MHC-I complex using computational resources available through the Vanderbilt Advanced Computing Center for Research and Education (ACCRE). The Rosetta energy composite score reweighted_sc, which provides additional weighting to interactions at the peptide-receptor interface, was averaged from the top 5 scoring models. PyMOL molecular graphics software was used to visualize the generated models (Schrödinger).

Candidate peptides 9 residues in length were derived from the sequences of proteins overexpressed in renal tissue. We queried the Human Protein Atlas for proteins with a greater than 4-fold increase in RNA expression in the kidney compared with other tissues, identified mouse homologs, and used NetMHCpan 4.0 to identify peptides derived from those sequences that were likely to bind strongly to H-2D^b^ (45).

### Peptide synthesis, IsoLG adduction, and in vitro IsoLG adduct generation.

Candidate peptides were commercially produced (EZBiolab). Peptides were N-terminally acetylated to prevent unwanted N-terminal IsoLG adduction, and purity confirmed with high-performance liquid chromatography. IsoLG was synthesized as described previously (74). Peptides were incubated with IsoLG at a 1:1 molar ratio of lysine to IsoLG overnight at 4°C in aqueous solution to generate adducts, as previously described (19). Unadducted and IsoLG-adducted peptides were stored at 4°C at a concentration of 1 mM until used in cell culture or flow cytometry.

Native IsoLG generation was induced by treating cultured cells with tBHP (Sigma-Adrich) at 100 μM for 30 minutes, as previously described (23). After treatment, cells were centrifuged at 350*g* for 5 minutes and the media replaced. Cells were incubated overnight prior to further analysis.

### Cell isolation and culture.

For assays with bead-bound MHC-I, spleens from H-2D^b^– or H-2K^b^–transgenic mice were isolated, passed through a 70-μm mesh, and red blood cells lysed (RBC lysis buffer, eBioscience) prior to culture in RPMI supplemented with 10% v/v fetal bovine serum, 1% v/v penicillin/streptomycin (Gibco), and 2 μL/500 mL of β-mercaptoethanol (Sigma-Aldrich). After 72 hours, media were collected and incubated with Ni-agarose beads (Thermo Fisher Scientific) and the beads collected through centrifugation before being added to T cell cultures. If indicated, a single-chain variable fragment antibody that recognizes IsoLG adducts (D11) was added to cultures simultaneously (D11 developed and produced as described in ref. 75).

For T cell proliferation assays, DCs were isolated from the spleens of C57BL/6 animals using the Miltenyi Biotec Pan Dendritic Cell Isolation Kit and cultured in RPMI supplemented with 10% v/v fetal bovine serum, 10 mM HEPES buffer, 1% v/v sodium pyruvate (Gibco), 1% v/v penicillin/streptomycin, and 2 μL/500 mL of β-mercaptoethanol. Adducted or unadducted peptides were added to DC cultures overnight at a concentration of 10 μM, DCs centrifuged, and supernatant removed prior to the addition of T cells. CD3^+^ cells were isolated from the bone marrow of adult C57BL/6 angiotensin II– or sham-treated mice using the Miltenyi Pan T Cell Isolation Kit II and cultured in RPMI supplemented with 10% v/v fetal bovine serum, 1% v/v MEM nonessential amino acids solution (Gibco), 1% v/v penicillin/streptomycin, and 0.4 μL/100 mL of β-mercaptoethanol. T cells were stained with CellTrace CFSE (Thermo Fisher Scientific) prior to incubation with DCs to track proliferation. A 1:1000 dilution of anti-CD28 (clone 37.51, BioLegend, 102101) was added to T cell and DC cocultures for costimulation. T cells were cultured for 5 days prior to analysis with flow cytometry as described above.

### Coimmunoprecipitation of IsoLG-adducted proteins.

Two milligrams of kidney protein was incubated overnight at 4°C with D11 antibody and then exposed to anti-FLAG M2 beads (Sigma-Aldrich, A2220) for 2 hours. After 2 washes in 0.1% Tween 20/PBS, the immunoprecipitated proteins were resolved by SDS-PAGE, transferred to nitrocellulose membranes, and immunoblotted with anti-CADH16 (clone CDH16/1532R, Abcam, ab270263) or anti-SGLT2 (clone EPR27112-7, Abcam, ab306558) antibodies.

### Tissue isolation and flow cytometry.

Femurs, tibias, aortas, and kidneys were isolated from sham- or angiotensin II–treated mice following perfusion of a normal saline solution to remove residual leukocytes from the peripheral blood. Long bones were flushed with PBS to isolate bone marrow cells. Aortas were manually homogenized and digested for 20 minutes at 37°C in RPMI with 1 mg/mL collagenase A and collagenase B and 0.1 mg/mL DNase I (Roche). Kidneys were homogenized using the Miltenyi Biotec gentleMACS Dissociator and digested for 20 minutes at 37°C in RPMI with 2 mg/mL collagenase D (Roche) and 0.1 mg/mL DNase I. Red blood cells were lysed for all tissues using RBC lysis buffer (eBioscience). Single-cell suspensions were stained with Live/Dead Violet fluorescent reactive dye (Thermo Fisher Scientific), Fc receptor block (anti-CD16/anti-CD32; clone S17011E, BioLegend, 156603), and stained with antibodies for CD3 (BV650; clone 17A2, BioLegend, 100229), CD4 (FITC; clone GK1.5, BioLegend, 100405), CD8a (APC; clone 53-6.7, BioLegend, 100711), CD44 (APC-Fire750; clone IM7, BioLegend, 103061), and CD62L (PE-Fire810; clone W18021D, BioLegend, 161205). To identify peptide-specific T cells, suspensions were also stained with H-2D^b^–IgG1 fusion protein (DimerX I:Mouse H-2D[b]:IgG; BD Biosciences, 551323) loaded with unadducted or IsoLG-adducted peptide (160:1 molar ratio overnight at 37°C) at 1 μg per sample. Secondary staining to identify H-2D^b^–IgG1–bound cells was done with anti-mouse IgG1 (PE; clone A85-1, BD Biosciences, 550083). Samples were run on a Cytek Aurora 4-laser flow cytometer and analyzed using FlowJo (Tree Star). A minimum of 1 × 10^6^ events were collected and cell counts determined based on total cell number (bone marrow and aortic cells, determined after isolation using a hemocytometer with trypan blue staining) or normalized to organ weight (kidney).

### Assessment of IsoLG adduct presentation by single-variant HLA–expressing cells.

We identified HLA alleles to screen for IsoLG adduct presentation by selecting the 10 HLA alleles with the highest phenotypic frequency present in 3 populations of the US National Merit Donor Program available through the Allele Frequency Net Database to best capture haplotype diversity (47). The populations included USA NMDP European Caucasian, USA NMDP Chinese, and USA NMDP African American Pop 2. Twenty-two nonduplicate HLA-A, -B, and -C alleles were identified in this manner.

The HLA-null human cell line K562 was transduced with each HLA allele with either an adenoviral vector or electroporation in conjunction with a transposon sequence and sleeping beauty transposase. After transduction and expansion, HLA-expressing cells were enriched by positive magnetic sorting using a biotinylated anti–pan-HLA antibody (clone W6/32, BioLegend, 311434) in conjunction with the CELLection Dynabead cell isolation kit (Thermo Fisher Scientific). HLA expression was confirmed with flow cytometry after staining with Live/Dead Violet fluorescent reactive dye and anti–pan-HLA (BV650). Nontransduced K562 cells were used as a null control.

To induce IsoLG adduct presentation, cells were treated with tBHP as described above. If indicated, cells were also maintained in media containing ethyl-2-HOBA at a concentration of 200 μM for the duration of the experiment. After incubation overnight, cells were stained with Live/Dead Violet fluorescent reactive dye and anti–pan-HLA (BV650) and biotinylated D11 conjugated to streptavidin APC-Cy7. D11 was biotinylated prior to conjugation with the streptavidin fluorophore using the Lightning Link A Biotinylation Kit (Abcam).

### Mass spectrometry of IsoLG-adducted peptides.

To confirm the presence of IsoLG modifications, IsoLG-reacted peptides were and analyzed by LC/MS on an orbitrap Exploris 480 instrument equipped with an Easy-nLC 1200 HPLC system, a PepMap C18 trapping column (75 μm × 2 cm), a PepMap RSLC C18 analytical column (75 μm × 15 cm), and an Easy-Spray ion source (Thermo Fisher Scientific). Peptides were separated by a 32-minute gradient with buffer A (0.1% formic acid) and buffer B (acetonitrile 80% with 0.1% formic acid) at 300 nL/min as follows: 5% to 10% by 2 minutes, 63% B by 22 minutes, 100% B by 24 minutes held until 32 minutes, and then reequilibrated at 0% B. Precursor ion scans were acquired with a resolution of 60,000 and product ions were scanned at 30,000. The precursor isolation window was *m*/*z* 0.7. A normalized collision energy of 30% was used in HCD mode. PRM precursor *m*/*z* values were chosen to correspond to the singly and doubly charged species of *N*-acetylated peptides with anhydropyrrole, pyrrole, anhydrolactam, or lactam modifications at lysine ε-amino groups. Data analysis was performed using Skyline 22.2.0.527 and Thermo Freestyle 1.8 SP2.

### Statistics.

Unless otherwise stated, data in the manuscript are presented as mean values ± standard deviation (SD). The 2-tailed Student’s *t* test was used for comparing means between 2 normally distributed data sets; otherwise, nonparametric tests were chosen. For normally distributed data with more than 2 mean values, 1-way or 2-way ANOVA was performed followed by appropriate post hoc tests; otherwise, nonparametric tests were used. Unless otherwise stated, differences between means were considered significant if the probability of the null hypothesis being true was less than 0.05.

### Study approval.

All animal experiments were approved by the Vanderbilt University Medical Center Institutional Animal Care and Use Committee.

### Data availability.

All code used to generate the models in this manuscript is provided, along with a detailed protocol capture illustrating the steps for reproducing these data, in a public GitHub repository (https://github.com/meilerlab/discovery-self-peptides-hypertension). The data presented in the figures and supplemental material are included in their entirety in the Supplemental Data Values file.

## Author contributions

NB designed the studies, conducted experiments, analyzed data, and wrote the manuscript. WC, KH, DP, AP, and MA analyzed data and conducted experiments. EP and SD provided reagents and wrote the manuscript. DR and MK conducted experiments, analyzed data, and wrote the manuscript. SM, RM, and JM designed studies and wrote the manuscript. DGH designed studies, analyzed data, and wrote the manuscript.

## Supplementary Material

Supplemental data

Supporting data values

## Version 1 08/15/2024

Electronic publication

## References

[1] Norlander AE (2018). The immunology of hypertension. J Exp Med.

[2] Madhur MS (2021). Hypertension: do inflammation and immunity hold the key to solving this epidemic?. Circ Res.

[3] Guzik TJ (2007). Role of the T cell in the genesis of angiotensin II induced hypertension and vascular dysfunction. J Exp Med.

[4] Rudemiller N (2014). CD247 modulates blood pressure by altering T-lymphocyte infiltration in the kidney. Hypertension.

[5] Itani HA (2016). Activation of human T cells in hypertension: studies of humanized mice and hypertensive humans. Hypertension.

[6] Youn J-C (2013). Immunosenescent CD8^+^ T cells and C-X-C chemokine receptor type 3 chemokines are increased in human hypertension. Hypertension.

[7] Nguyen H (2013). Interleukin-17 causes Rho-kinase-mediated endothelial dysfunction and hypertension. Cardiovasc Res.

[8] Madhur MS (2010). Interleukin 17 promotes angiotensin II-induced hypertension and vascular dysfunction. Hypertension.

[9] Trott DW (2014). Oligoclonal CD8^+^ T cells play a critical role in the development of hypertension. Hypertension.

[10] James EA (2014). Citrulline-specific Th1 cells are increased in rheumatoid arthritis and their frequency is influenced by disease duration and therapy. Arthritis Rheumatol.

[11] Sharma RK (2021). Biased TCR gene usage in citrullinated Tenascin C specific T-cells in rheumatoid arthritis. Sci Rep.

[12] Pavlos R (2015). T cell-mediated hypersensitivity reactions to drugs. Annu Rev Med.

[13] Monshi MM (2013). Human leukocyte antigen (HLA)-B*57:01-restricted activation of drug-specific T cells provides the immunological basis for flucloxacillin-induced liver injury. Hepatology.

[14] Kacen A (2023). Post-translational modifications reshape the antigenic landscape of the MHC I immunopeptidome in tumors. Nat Biotechnol.

[15] Salomon RG, Miller DB (1985). Levuglandins: isolation, characterization, and total synthesis of new secoprostanoid products from prostaglandin endoperoxides. Adv Prostaglandin Thromboxane Leukot Res.

[16] Brame CJ (1999). Identification of extremely reactive gamma-ketoaldehydes (isolevuglandins) as products of the isoprostane pathway and characterization of their lysyl protein adducts. J Biol Chem.

[17] (1999). New developments in the isoprostane pathway: identification of novel highly reactive gamma-ketoaldehydes (isolevuglandins) and characterization of their protein adducts. FASEB J.

[18] Iyer RS (1989). Levuglandin E2 crosslinks proteins. Prostaglandins.

[19] Patrick DM (2022). Isolevuglandins disrupt PU.1-mediated C1q expression and promote autoimmunity and hypertension in systemic lupus erythematosus. JCI Insight.

[20] Prinsen JK (2020). Highly reactive isolevuglandins promote atrial fibrillation caused by hypertension. JACC Basic Transl Sci.

[21] Tao H (2020). Scavenging of reactive dicarbonyls with 2-hydroxybenzylamine reduces atherosclerosis in hypercholesterolemic Ldlr^-/-^ mice. Nat Commun.

[22] Ngwenyama N (2021). Isolevuglandin-modified cardiac proteins drive CD4^+^ T-cell activation in the heart and promote cardiac dysfunction. Circulation.

[23] Annet K (2014). DC isoketal-modified proteins activate T cells and promote hypertension. J Clin Invest.

[24] Vinh A (2010). Inhibition and genetic ablation of the B7/CD28 T-cell costimulation axis prevents experimental hypertension. Circulation.

[25] Xiao L (2020). Sympathetic enhancement of memory T-cell homing and hypertension sensitization. Circ Res.

[26] Ruggeri Barbaro N (2021). Sodium activates human monocytes via the NADPH oxidase and isolevuglandin formation. Cardiovasc Res.

[27] Van Beusecum JP (2019). High salt activates CD11c^+^ antigen-presenting cells via SGK (serum glucocorticoid kinase) 1 to promote renal inflammation and salt-sensitive hypertension. Hypertension.

[28] Van Beusecum JP (2021). Growth arrest specific-6 and Axl coordinate inflammation and hypertension. Circ Res.

[29] Archbold JK (2009). Natural micropolymorphism in human leukocyte antigens provides a basis for genetic control of antigen recognition. J Exp Med.

[30] Illing PT (2012). Immune self-reactivity triggered by drug-modified HLA-peptide repertoire. Nature.

[31] Chen B (2013). Association of ankylosing spondylitis with HLA-B27 and ERAP1: pathogenic role of antigenic peptide. Med Hypotheses.

[32] Boisgerault F (1996). Definition of the HLA-A29 peptide ligand motif allows prediction of potential T-cell epitopes from the retinal soluble antigen, a candidate autoantigen in birdshot retinopathy. Proc Natl Acad Sci U S A.

[33] Nair RP (2006). Sequence and haplotype analysis supports HLA-C as the psoriasis susceptibility 1 gene. Am J Hum Genet.

[34] Scally SW (2013). A molecular basis for the association of the HLA-DRB1 locus, citrullination, and rheumatoid arthritis. J Exp Med.

[35] Petersen J (2014). T-cell receptor recognition of HLA-DQ2-gliadin complexes associated with celiac disease. Nat Struct Mol Biol.

[36] Young AC (1994). The three-dimensional structure of H-2Db at 2.4 A resolution: implications for antigen-determinant selection. Cell.

[37] Fremont DH (1995). Crystal structure of an H-2Kb-ovalbumin peptide complex reveals the interplay of primary and secondary anchor positions in the major histocompatibility complex binding groove. Proc Natl Acad Sci U S A.

[38] Achour A (2002). A structural basis for LCMV immune evasion: subversion of H-2D(b) and H-2K(b) presentation of gp33 revealed by comparative crystal structure.Analyses. Immunity.

[39] Raveh B (2010). Sub-angstrom modeling of complexes between flexible peptides and globular proteins. Proteins.

[40] Tengfei L (2014). Subangstrom accuracy in pHLA-I modeling by Rosetta FlexPepDock refinement protocol. J Chem Inf Model.

[41] Alam N, Schueler-Furman O (2017). Modeling peptide-protein structure and binding using Monte Carlo sampling approaches: Rosetta FlexPepDock and FlexPepBind. Methods Mol Biol.

[42] Vita R (2015). The immune epitope database (IEDB) 3.0. Nucleic Acids Res.

[43] Sigal LJ, Wylie DE (1996). Role of non-anchor residues of Db-restricted peptides in class I binding and TCR triggering. Mol Immunol.

[44] Uhlén M (2015). Proteomics. Tissue-based map of the human proteome. Science.

[45] Hoof I (2009). NetMHCpan, a method for MHC class I binding prediction beyond humans. Immunogenetics.

[46] Itani HA (2016). CD70 exacerbates blood pressure elevation and renal damage in response to repeated hypertensive stimuli. Circ Res.

[47] Gonzalez-Galarza FF (2020). Allele frequency net database (AFND) 2020 update: gold-standard data classification, open access genotype data and new query tools. Nucleic Acids Res.

[48] Wu J (2016). Immune activation caused by vascular oxidation promotes fibrosis and hypertension. J Clin Invest.

[49] McDonnell WJ (2018). High CD8 T-cell receptor clonality and altered CDR3 properties are associated with elevated isolevuglandins in adipose tissue during diet-induced obesity. Diabetes.

[50] Fernandez DM (2019). Single-cell immune landscape of human atherosclerotic plaques. Nat Med.

[51] Winkels H (2018). Atlas of the immune cell repertoire in mouse atherosclerosis defined by single-cell RNA-sequencing and mass cytometry. Circ Res.

[52] Sun S (2019). Gut microbiota composition and blood pressure. Hypertension.

[53] Karbach SH (2016). Gut microbiota promote angiotensin II-induced arterial hypertension and vascular dysfunction. J Am Heart Assoc.

[54] Wilck N (2017). Salt-responsive gut commensal modulates T_H_17 axis and disease. Nature.

[55] Elijovich F (2020). The gut microbiome, inflammation, and salt-sensitive hypertension. Curr Hypertens Rep.

[56] Massey KJ (2012). Angiotensin II stimulates superoxide production in the thick ascending limb by activating NOX4. Am J Physiol Cell Physiol.

[57] Welch WJ (2005). Angiotensin-induced defects in renal oxygenation: role of oxidative stress. Am J Physiol Heart Circ Physiol.

[58] Boutaud O (1999). Characterization of the lysyl adducts formed from prostaglandin H2 via the levuglandin pathway. Biochemistry.

[59] Dendrou CA (2018). HLA variation and disease. Nat Rev Immunol.

[60] Diamantopoulos EJ (2003). HLA phenotypes as promoters of cardiovascular remodelling in subjects with arterial hypertension. J Hum Hypertens.

[61] Gerbase-DeLima M (1992). Essential hypertension and histocompatibility antigens. An association study. Hypertension.

[62] Karnes JH (2017). Phenome-wide scanning identifies multiple diseases and disease severity phenotypes associated with HLA variants. Sci Transl Med.

[63] Harrison DG (2021). Pathophysiology of hypertension: the mosaic theory and beyond. Circ Res.

[64] Bouvier M, Wiley DC (1994). Importance of peptide amino and carboxyl termini to the stability of MHC class I molecules. Science.

[65] Yu F (2020). Identification of modified peptides using localization-aware open search. Nat Commun.

[66] Achar SR (2022). Universal antigen encoding of T cell activation from high-dimensional cytokine dynamics. Science.

[67] François P, Altan-Bonnet G (2016). The case for absolute ligand discrimination: modeling information processing and decision by immune T cells. J Stat Phys.

[68] Caillon A (2017). γδ T cells mediate angiotensin II-induced hypertension and vascular injury. Circulation.

[69] Xue B (2005). Sex differences in the development of angiotensin II-induced hypertension in conscious mice. Am J Physiol Heart Circ Physiol.

[70] Sandberg K, Ji H (2012). Sex differences in primary hypertension. Biol Sex Differ.

[71] Bloodworth N (2022). Rosetta FlexPepDock to predict peptide-MHC binding: an approach for non-canonical amino acids. PLoS One.

[72] Fleishman SJ (2011). RosettaScripts: a scripting language interface to the rosetta macromolecular modeling suite. PLoS One.

[73] Jumper J (2021). Highly accurate protein structure prediction with AlphaFold. Nature.

[74] Amarnath V (2005). A simplified synthesis of the diastereomers of levuglandin E_2_. Synth Commun.

[75] Davies SS (2004). Localization of isoketal adducts in vivo using a single-chain antibody. Free Radic Biol Med.

