# Supplementary material for: Posttranslationally modified self-peptides promote hypertension in mouse models
Source: J Clin Invest. 2024 Aug 15;134(16):e174374. doi: 10.1172/JCI174374 (PMC11324298; doi:10.1172/JCI174374)
Supplement: Supplemental data [file jci-134-174374-s245.pdf]

H-2D^b (912 bp)

```
atgggggcgatggctccgcgcacgctgctcctgctgctggcggccgccctggccccgactcag
acccgcgcgggcccacactcgatgcggtatttcgagaccgccgtgtcccggcccggcctcgag
gagccccggtacatctctgtcggctatgtggacaacaaggagttcgtgcgcttcgacagcgac
gcggagaatccgagatatgagccgcgggcgccgtggatggagcaggagggccggagtattgg
gagcgggaaacacagaaagccaagggccaagagcagtggttccgagtgagcctgaggaacctg
ctcggctactacaaccagagcgcgggcggctctcacacactccagcagatgtctggctgtgac
ttggggtcggactggcgcctcctccgcgggtacctgcagttcgcctatgaaggccgcgattac
atcgccctgaacgaagacctgaaaacgtggacggcggcggacatggcggcgcagatcacccga
cgcaagtgggagcagagtggtgctgcagagcattacaaggcctacctggagggcgagtgcgtg
gagtggctccacagatacctgaagaacgggaacgcgacgctgctgcgcacagattccccaaag
gcacatgtgacccatcaccccagatctaaaggtgaagtcaccctgaggtgctgggccctgggc
ttctaccctgctgacatcaccctgacctggcagttgaatggggaggagctgacccaggacatg
gagcttgtggagaccaggcctgcaggggatggaaccttccagaagtgggcatctgtggtggtg
cctcttgggaaggagcagaattacacatgccgtgtgtaccatgaggggctgcctgagcccctc
accctgagatgggagcctcctccgtccact
```

H-2K^b (840 bp)

```
ggcccacactcgctgaggtatttcgtcaccgccgtgtcccggcccggcctcggggagccccgg
tacatggaagtcggctacgtggacgacacggagttcgtgcgcttcgacagcgacgcggagaat
ccgagatatgagccgcgggcgcggtggatggagcaggagggcccgagtattgggagcgggag
acacagaaagccaagggcaatgagcagagtttccgagtggacctgaggaccctgctcggctac
tacaaccagagcaagggcggctctcacactattcaggtgatctctggctgtgaagtggggtcc
gacgggcgactcctccgcgggtaccagcagtacgcctacgacggctgcgattacatcgccctg
aacgaagacctgaaaacgtggacggcggcggacatggcggcgctgatcaccaaacacaagtgg
gagcaggctggtgaagcagagagactcagggcctacctggagggcacgtgcgtggagtggctc
cgcagatacctgaagaacgggaacgcgacgctgctgcgcacagattccccaaaggcccatgtg
acccatcacagcagacctgaagataaagtcaccctgaggtgctgggccctgggcttctaccct
gctgacatcaccctgacctggcagttgaatggggaggagctgatccaggacatggagcttgtg
gagaccaggcctgcaggggatggaaccttccagaagtgggcatctgtggtggtgcctcttggg
aaggagcagtattacacatgccatgtgtaccatcaggggctgcctgagcccctcaccctgaga
tgggagcctcctccatccact
```

Supplemental Table 1: Soluble MHC-I heavy chain sequences for H-2D^b and H-2K^b

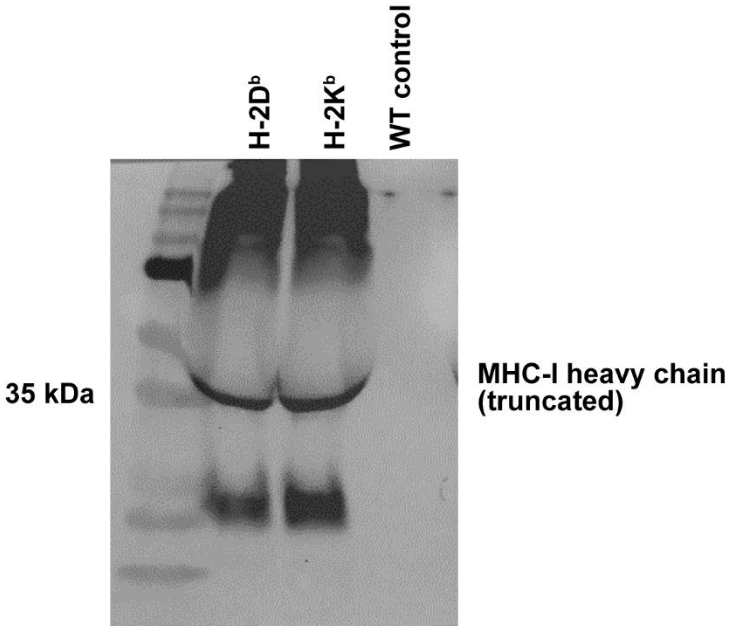

Supplemental Figure 1: Soluble His-tagged MHC-I is present in the media of cultured splenocytes isolated from transgenic mice. His-tagged proteins were immunoprecipitated and separated by gel electrophoresis. Staining with antibodies for MHC-I confirms the presence of soluble MHC-I.

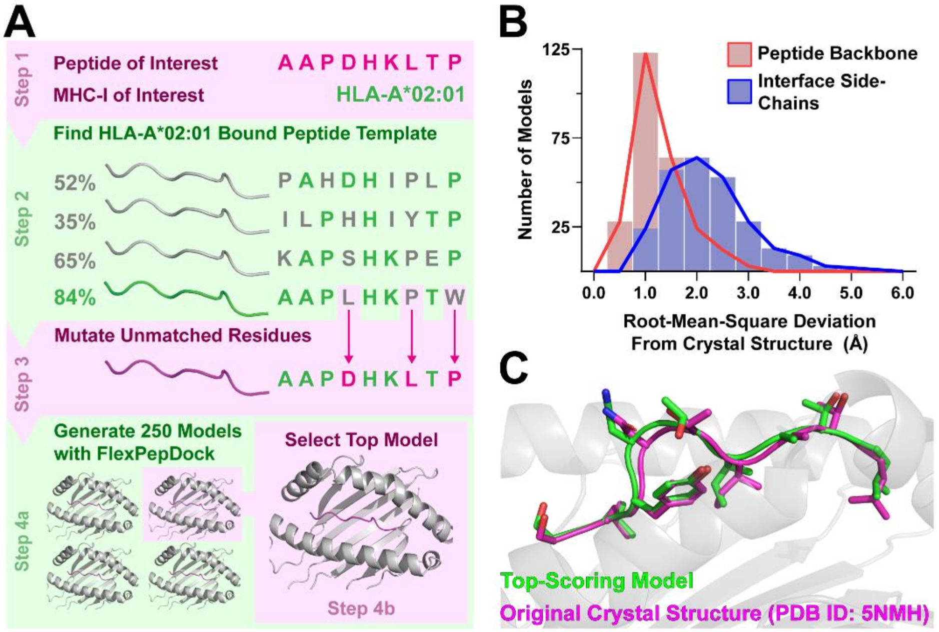

Supplemental Figure 2: FlexPepDock refinement can accurately recapitulate MHC-I bound peptide structures using pre-existing templates. (A) Diagram illustrating our approach for selecting peptide templates from the PDB based on sequence similarity. (B) A benchmark on available MHC-I molecules in the PDB with a resolution <2.5 Å yields structures with low root-mean-square deviation (RMSD) from the original crystal structures (n=329). Heavy atom side-chain RMSD was also <2.5 for most structures generated. (C) Example of a peptide model generated by FlexPepDock refinement (PDB ID 5NMH. Model shown in green, original structure in magenta).

			Template		RMSD	
**PDB**	**MHC-I**	**Epitope**	**PDB**	**MHC-I**	**Backbone**	**Interface**
1A1M	B*53:01	TPYDINQML	1A1O	B*53:01	1.35	2.59
1A1N	B*35:01	VPLRPMTY	3BWA	B*35:08	1.73	2.48
1A1O	B*53:01	KPIVQYDNF	1A1M	B*53:01	1.08	2.94
1A9E	B*35:01	LPPLDITPY	4QRR	B*35:01	1.98	3.24
1AGB	B*08:01	GGRKKYKL	1AGD	B*08:01	0.79	1.18
1AGC	B*08:01	GGKKKYQL	1AGD	B*08:01	0.40	1.33
1AGD	B*08:01	GGKKKYKL	1AGE	B*08:01	0.41	1.55
1AGE	B*08:01	GGKKKYRL	1AGD	B*08:01	0.36	1.47
1AGF	B*08:01	GGKKRYKL	1AGD	B*08:01	0.45	1.43
1DUY	A*02:01	LFGYPVYV	4F7T	A*24:02	3.06	4.63
1DUZ	A*02:01	LLFGYPVYV	3QFJ	A*02:01	1.47	1.87
1E27	B*51:01	LPPVVAKEI	1A1M	B*53:01	2.77	4.14
1EEY	A*02:01	ILSALVGIV	1EEZ	A*02:01	1.37	1.95
1EEZ	A*02:01	ILSALVGIL	1EEY	A*02:01	1.32	1.83
1EFX	C*03:04	GAVDPLLAL	4NT6	C*08:01	2.27	3.59
1HHG	A*02:01	TLTSCNTSV	7N1E	A*02:01	1.60	2.22
1HHH	A*02:01	FLPSDFFPSV	5C0G	A*02:01	2.21	3.43
1HSA	B*27:05	ARAAAAAAA	1JGE	B*27:05	1.52	1.67
1I1F	A*02:01	FLKEPVHGV	1I1Y	A*02:01	0.90	1.90
1I1Y	A*02:01	YLKEPVHGV	1I1F	A*02:01	1.17	2.58
1I4F	A*02:01	GVYDGREHTV	6TRN	A*02:01	0.82	2.63
1I7R	A*02:01	FAPGFFPYL	1LP9	A*02:01	1.13	1.72
1I7T	A*02:01	ALWGVFPVL	1LP9	A*02:01	0.79	1.01
1I7U	A*02:01	ALWGFVPVL	1LP9	A*02:01	1.38	2.12
1JF1	A*02:01	ELAGIGILTV	4JFE	A*02:01	0.82	1.55
1JGE	B*27:05	GRFAAAIAK	1HSA	B*27:05	0.77	1.64
1LP9	A*02:01	ALWGFFPVL	1I7U	A*02:01	1.00	1.45
1M05	B*08:01	FLRGRAYGL	3SKM	B*08:01	1.84	2.06
1M6O	B*44:02	EEFGRAFSF	3L3G	B*44:02	0.59	1.34
1N2R	B*44:03	EEFGRAFSF	3KPN	B*44:03	1.46	3.32
1OGA	A*02:01	GILGFVFTL	5HHQ	A*02:01	1.56	2.30
1Q94	A*11:01	AIFQSSMTK	6JOZ	A*11:01	1.50	2.23
1QQD	C*04:01	QYDDAVYKL	5VGD	C*05:01	1.16	2.31
1QRN	A*02:01	LLFGYAVYV	1DUZ	A*02:01	1.09	2.95
1QSE	A*02:01	LLFGYPRYV	1DUZ	A*02:01	1.20	1.57
1QSF	A*02:01	LLFGYPVAV	1DUZ	A*02:01	1.06	2.61
1QVO	A*11:01	QVPLRPMTYK	5WKF	A*11:01	1.98	3.23
1S8D	A*02:01	SLANTVATL	2V2X	A*02:01	1.45	2.59
1S9X	A*02:01	SLLMWITQA	1S9Y	A*02:01	1.39	2.29
1S9Y	A*02:01	SLLMWITQS	1S9X	A*02:01	0.99	1.80
1SYS	B*44:03	EEPTVIKKY	3KPN	B*44:03	1.94	2.16
1SYV	B*44:05	EEFGRAFSF	3KPP	B*44:05	1.65	3.34
1T1X	A*02:01	SLYLTVATL	2V2W	A*02:01	0.98	1.75
1T1Y	A*02:01	SLYNVVATL	2V2W	A*02:01	0.96	1.40
1T1Z	A*02:01	ALYNTAAAL	2V2W	A*02:01	0.81	1.29
1TVB	A*02:01	ITDQVPFSV	1TVH	A*02:01	0.71	2.03
1TVH	A*02:01	IMDQVPFSV	6VMC	A*02:01	0.79	2.54
1UXS	B*27:05	RRRWRRLTV	5IB2	B*27:05	1.38	2.53
1W0V	B*27:05	RRLPIFSRL	2BSR	B*27:05	1.09	1.93
1X7Q	A*11:01	KTFPPTEPK	1Q94	A*11:01	2.04	3.27
1XR8	B*15:01	LEKARGSTY	1XR9	B*15:01	0.96	2.97
1XR9	B*15:01	ILGPPGSVY	1XR8	B*15:01	1.76	2.26
2A83	B*27:05	RRRWHRWRL	5IB2	B*27:05	1.01	2.47
2AXF	B*35:08	APQPAPENAY	5VZ5	B*15:01	2.77	3.65
2AXG	B*35:01	APQPAPENAY	5VZ5	B*15:01	2.54	3.46
2BCK	A*24:02	VYGFVRACL	6XQA	A*24:02	1.07	1.67
2BNQ	A*02:01	SLLMWITQV	3KLA	A*02:01	0.91	2.63
2BSR	B*27:05	RRIYDLIEL	1W0V	B*27:05	1.26	2.38
2BST	B*27:05	SRYWAIRTR	3LV3	B*27:05	1.02	3.09
2BVP	B*57:03	ISPRTLNAW	5VVP	B*57:03	1.44	2.25
2BVQ	B*57:03	KAFSPEVI	3W39	B*52:01	0.98	1.60
2CIK	B*35:01	KPIVVLHGY	4QRR	B*35:01	1.43	2.68
2CLR	A*02:01	MLLSVPLLLG	4JFP	A*02:01	2.82	3.92
2GT9	A*02:01	EAAGIGILTV	1JF1	A*02:01	1.03	1.48
2GTW	A*02:01	LAGIGILTV	3QFD	A*02:01	2.86	3.31
2GTZ	A*02:01	ALGIGILTV	3QFD	A*02:01	1.22	1.83
2HN7	A*11:01	AIMPARFYPK	5WKF	A*11:01	2.09	3.87
2P5E	A*02:01	SLLMWITQC	1S9X	A*02:01	0.81	2.05
2V2W	A*02:01	SLYNTVATL	5NMH	A*02:01	0.87	1.49

			Template		RMSD	
**PDB**	**MHC-I**	**Epitope**	**PDB**	**MHC-I**	**Backbone**	**Interface**
2X4O	A*02:01	KLTPLCVTL	3MRC	A*02:01	1.58	2.60
2X4S	A*02:01	AMDSNTLEL	3D25	A*02:01	1.25	2.40
2X4U	A*02:01	ILKEPVHGV	1I1F	A*02:01	0.94	1.49
2XPG	A*03:01	KLIETYFSK	3RL1	A*03:01	1.05	2.50
3BO8	A*01:01	EADPTGHSY	5BRZ	A*01:01	0.75	1.33
3BP4	B*27:05	IRAAPPPLF	1HSA	B*27:05	1.75	2.74
3BWA	B*35:08	FPTKDVAL	1A1N	B*35:01	0.81	1.47
3BXN	B*14:02	IRAAPPPLF	4O2E	B*39:01	2.43	3.26
3C9N	B*15:01	VQQESSFVM	1XR8	B*15:01	1.07	1.97
3D25	A*02:01	VLHDDLLEA	3FT4	A*02:01	0.86	1.66
3DX6	B*44:02	EENLLDFVRF	2AXF	B*35:08	1.42	2.67
3DX7	B*44:03	EENLLDFVRF	2AXG	B*35:01	2.02	4.28
3DX8	B*44:05	EENLLDFVRF	2AXF	B*35:08	2.25	4.54
3FQT	A*02:01	GLLGSPVRA	1QSF	A*02:01	1.50	2.92
3FT4	A*02:01	VLRDDLLEA	3D25	A*02:01	0.76	1.60
3GIV	A*02:01	SLFNTVATLY	3MRN	A*02:01	2.23	5.27
3GSQ	A*02:01	NLVPSVATV	3GSU	A*02:01	0.84	1.32
3GSR	A*02:01	NLVPVVATV	6Q3K	A*02:01	0.82	1.61
3GSU	A*02:01	NLVPTVATV	3GSQ	A*02:01	0.82	1.22
3GSV	A*02:01	NLVPQVATV	3GSQ	A*02:01	0.86	1.68
3GSW	A*02:01	NLVPMVAAV	3GSX	A*02:01	0.83	1.85
3GSX	A*02:01	NLVPMVAVV	3GSW	A*02:01	0.86	1.58
3H7B	A*02:01	MLWGYLQYV	1QSE	A*02:01	0.90	1.45
3HPJ	A*02:01	RMFPNAPYL	1QRN	A*02:01	1.05	2.87
3I6G	A*02:01	GLMWLSYFV	5HHN	A*02:01	0.99	1.85
3I6K	A*02:01	TLACFVLAAV	4JFQ	A*02:01	1.89	2.71
3KLA	A*02:01	SLLMWITQL	2BNQ	A*02:01	1.22	2.29
3KPL	B*44:02	EEYLQAFTY	3KPM	B*44:02	1.40	2.75
3KPM	B*44:02	EEYLKAWTF	3KPL	B*44:02	1.28	3.12
3KPN	B*44:03	EEYLQAFTY	3KPO	B*44:03	1.69	2.88
3KPO	B*44:03	EEYLKAWTF	3KPN	B*44:03	1.71	3.62
3KPP	B*44:05	EEYLQAFTY	3KPQ	B*44:05	1.33	2.62
3KPQ	B*44:05	EEYLKAWTF	3KPP	B*44:05	1.45	3.15
3L3D	B*44:02	EEAGRAFSF	1M6O	B*44:02	0.66	2.00
3L3G	B*44:02	EEFGAAFSF	1M6O	B*44:02	0.87	1.81
3L3I	B*44:02	EEFGRAASF	1M6O	B*44:02	0.87	2.33
3L3J	B*44:02	EEAGAAFSF	3L3D	B*44:02	1.11	1.69
3L3K	B*44:02	EEFGAAASF	3L3I	B*44:02	1.50	1.63
3LKN	B*35:01	LPFERATIM	3LKR	B*35:01	0.95	1.60
3LKO	B*35:01	LPFDRTTIM	3LKQ	B*35:01	0.68	2.03
3LKP	B*35:01	LPFDKSTIM	3LKQ	B*35:01	0.90	1.87
3LKQ	B*35:01	LPFDKTTIM	3LKO	B*35:01	0.73	1.06
3LKR	B*35:01	LPFERATVM	3LKN	B*35:01	0.73	1.28
3LKS	B*35:01	LPFEKSTVM	3LKP	B*35:01	0.86	1.32
3LV3	B*27:05	SRRWRRWNR	5IB2	B*27:05	1.44	4.15
3MGO	A*02:01	RLYQNPTTYI	3MGT	A*02:01	1.65	2.96
3MGT	A*02:01	KLYQNPTTYI	3MGO	A*02:01	1.99	3.08
3MR9	A*02:01	NLVPAVATV	3GSQ	A*02:01	0.82	1.30
3MRB	A*02:01	NLVPMVHTV	6Q3K	A*02:01	0.95	1.38
3MRC	A*02:01	NLVPMCATV	6Q3K	A*02:01	1.93	2.72
3MRD	A*02:01	NLVPMGATV	3MRC	A*02:01	1.34	1.70
3MRE	A*02:01	GLCTLVAML	3GSX	A*02:01	1.60	2.06
3MRG	A*02:01	CINGVCWTV	3MRJ	A*02:01	1.54	2.25
3MRH	A*02:01	CISGVCWTV	3MRG	A*02:01	0.91	2.24
3MRI	A*02:01	CINMWCWTV	3MRJ	A*02:01	1.43	3.73
3MRJ	A*02:01	CINGMCWTV	3MRG	A*02:01	1.02	1.88
3MRK	A*02:01	PLFQVPEPV	6VMC	A*02:01	1.24	2.54
3MRL	A*02:01	CINGVVWTV	3MRG	A*02:01	0.99	2.26
3MRM	A*02:01	KLVALGINAV	3MRP	A*02:01	1.18	1.79
3MRN	A*02:01	LLFNILGGWV	3GIV	A*02:01	2.76	4.91
3MRO	A*02:01	ELAGWGILTV	1JF1	A*02:01	0.98	2.28
3MRP	A*02:01	ELAGLGINTV	4JFQ	A*02:01	0.94	1.62
3MRR	A*02:01	LLAGIGTVPI	6AM5	A*02:01	1.26	1.77
3OX8	A*02:03	FLPSDFFPSV	5C0G	A*02:01	1.61	3.36
3OXR	A*02:06	FLPSDFFPSV	5C0G	A*02:01	2.12	4.13
3OXS	A*02:07	FLPSDFFPSV	5C0G	A*02:01	1.93	3.68
3PWJ	A*02:01	LLYGFVNYV	3PWN	A*02:01	1.05	1.49
3PWL	A*02:01	LGYGFVNYI	3PWN	A*02:01	1.01	2.15
3PWN	A*02:01	LLYGFVNYI	3PWJ	A*02:01	0.89	1.33

			Template		RMSD	
PDB	MHC-I	Epitope	PDB	MHC-I	Backbone	Interface
3QFJ	A*02:01	LLFGFPVYV	1DUZ	A*02:01	0.91	2.47
3REW	A*02:01	CLGGLLTMV	3MRL	A*02:01	1.57	2.28
3RL1	A*03:01	AIFQSSMTK	7L1C	A*03:01	1.11	2.34
3RL2	A*03:01	QVPLRPMTYK	5WKF	A*11:01	2.77	4.00
3SKM	B*08:01	FLRGRAYVL	1M05	B*08:01	1.89	3.08
3SPV	B*08:01	RAKFKQLL	1AGD	B*08:01	0.53	1.07
3UPR	B*57:01	HSITYLLPV	5U98	B*57:01	1.20	2.22
3UTQ	A*02:01	ALWGPDPAAA	5C0D	A*02:01	1.16	1.46
3VH8	B*57:01	LSSPVTKSF	5VUD	B*57:01	1.14	1.24
3VRJ	B*57:01	LTTKLTNTNI	5T6X	B*57:01	3.42	5.10
3VXN	A*24:02	RYPLTFGWCF	5HGD	A*24:02	1.52	2.23
3VXS	A*24:02	RYPLTLGWCF	3VXN	A*24:02	1.06	1.60
3W39	B*52:01	TAFTIPSI	2BVQ	B*57:03	1.24	2.13
3WL9	A*24:02	NYTPGPGIRF	3WLB	A*24:02	0.90	1.41
3WLB	A*24:02	NYTPGPGTRF	3WL9	A*24:02	1.99	1.59
4F7M	A*24:02	LYASPQLEGF	7JYU	A*24:02	1.06	2.05
4F7T	A*24:02	RYGFVANF	5HGB	A*24:02	0.98	1.75
4G8I	B*27:05	KRWIIMGLNK	4G9D	B*27:05	1.29	1.31
4G9D	B*27:05	KRWIILGLNK	4G8I	B*27:05	2.89	4.09
4GKN	A*02:01	FATGIGIITV	4GKS	A*02:01	1.65	2.54
4GKS	A*02:01	FLTGIGIITV	4GKN	A*02:01	1.25	2.16
4HWZ	A*68:01	AIFQSSMTK	4HX1	A*68:02	1.56	2.48
4HX1	A*68:02	SVYDFFVWL	4I48	A*68:02	1.30	2.57
4I48	A*68:02	TLTSCNTSV	4HX1	A*68:02	1.90	2.37
4I4W	A*02:01	ILAKFLHWL	5MEP	A*02:01	1.10	1.77
4JFE	A*02:01	ELAGIGALTV	1JF1	A*02:01	0.93	1.48
4JFO	A*02:01	ALAGIGILTV	1JF1	A*02:01	0.82	1.70
4JFP	A*02:01	ELAAIGILTV	1JF1	A*02:01	0.99	1.51
4JFQ	A*02:01	ELAGIGIATV	1JF1	A*02:01	1.04	1.54
4JQV	B*18:01	SELEIKRY	4XXC	B*18:01	0.71	1.26
4K7F	A*02:01	VCWGELMNL	3H7B	A*02:01	0.80	1.66
4L3C	A*02:01	YLLMWITQV	2BNQ	A*02:01	1.46	2.50
4MJI	B*51:01	TAFTIPSI	1A1N	B*35:01	1.32	2.51
4N8V	A*11:01	MLIYSMWGK	1Q94	A*11:01	1.86	2.97
4NNY	A*02:01	RQASLSISV	4NO5	A*02:01	1.98	2.97
4NO5	A*02:01	RQISQDVKL	4NNY	A*02:01	1.84	2.89
4NQV	A*01:01	CTELKLSDY	4NQX	A*01:01	0.66	1.40
4NQX	A*01:01	CTELKLNDY	4NQV	A*01:01	0.74	1.74
4NT6	C*08:01	GILGFVFTL	6ULI	C*08:02	1.85	3.70
4O2E	B*39:01	SHVAVENAL	3BXN	B*14:02	2.45	3.90
4O2F	B*39:01	HVAVENAL	3SPV	B*08:01	3.14	4.70
4QRQ	B*08:01	HSKKKCDEL	5WMR	B*08:01	2.21	2.44
4QRR	B*35:01	IPSINVHHY	1A9E	B*35:01	1.57	2.82
4QRS	B*08:01	ELKRKMIYM	4QRT	B*08:01	2.03	2.97
4QRT	B*08:01	ELNRKMIYM	4QRS	B*08:01	1.04	1.72
4QRU	B*08:01	ELRRKMMYM	4QRS	B*08:01	2.30	2.79
4U1H	B*07:02	TPQDLNTML	5EO0	B*07:02	0.58	1.12
4U1J	B*42:01	TPQDLNTML	4U1M	B*42:01	1.57	3.02
4U1K	B*07:02	RPQVPLRPM	5WMO	B*07:02	1.76	3.59
4U1M	B*42:01	RPQVPLRPM	4U1J	B*42:01	1.40	2.43
4XXC	B*18:01	DELEIKAY	4JQV	B*18:01	0.58	1.29
5BRZ	A*01:01	EVDPIGHLY	3BO8	A*01:01	1.48	1.90
5BS0	A*01:01	ESDPIVAQY	5BRZ	A*01:01	0.60	1.28
5C0D	A*02:01	AQWGPDPAAA	3UTQ	A*02:01	1.20	1.98
5C0E	A*02:01	YQFGPDFPIA	5C0I	A*02:01	0.95	2.30
5C0F	A*02:01	RQWGPDPAAV	5C0D	A*02:01	1.14	1.91
5C0G	A*02:01	YLGGPDFPTI	5C0I	A*02:01	1.05	1.65
5C0I	A*02:01	RQFGPDFPTI	5C0E	A*02:01	0.66	1.88
5C0J	A*02:01	RQFGPDWIVA	5C0E	A*02:01	1.40	2.37
5DEF	B*27:04	RRKWRRWHL	2A83	B*27:05	2.49	4.89
5E00	A*02:01	GVWIRTPPA	5WSH	A*02:01	0.82	1.77
5ENW	A*02:01	GLKEGIPAL	5FA3	A*02:01	0.65	1.56
5EO0	B*07:02	RPMTFKGAL	5EO1	B*07:02	0.62	1.70
5EO1	B*07:02	RPMTYKGAL	5EO0	B*07:02	0.64	1.39
5EU3	A*02:01	YLEPGPVTA	5EU6	A*02:01	1.14	2.15
5EU4	A*02:01	YLAPGPVTA	5EU3	A*02:01	1.63	2.20
5EU5	A*02:01	YLEPAPVTA	5EU3	A*02:01	1.11	2.13
5EU6	A*02:01	YLEPGPVTV	5EU3	A*02:01	1.24	2.41
5F9J	A*02:01	YLSPIASPL	7N1A	A*02:01	1.32	2.11

			Template		RMSD	
PDB	MHC-I	Epitope	PDB	MHC-I	Backbone	Interface
5FA3	A*02:01	GLLPELPAV	5ENW	A*02:01	0.66	1.19
5FDW	A*02:01	YLSPIASPLL	5C0G	A*02:01	1.97	2.73
5GRD	A*11:01	SSCSSCPLSK	5WJN	A*11:01	1.30	1.66
5GSD	A*11:01	SSCPLSK	6PBH	A*68:01	2.65	3.13
5HGA	A*24:02	RFPLTFGW	5HGB	A*24:02	1.24	1.78
5HGB	A*24:02	RYPLTFGW	5HGA	A*24:02	1.43	2.00
5HGD	A*24:02	RFPLTFGWCF	3VXN	A*24:02	1.17	1.82
5HHN	A*02:01	GILGLVFTL	1OGA	A*02:01	0.91	1.65
5HHP	A*02:01	GILEFVFTL	1OGA	A*02:01	0.92	3.00
5HHQ	A*02:01	GIWGFVFTL	1OGA	A*02:01	0.84	2.74
5IB2	B*27:05	RRKWRRWHL	2A83	B*27:05	1.16	3.61
5IEK	B*40:02	REFSKEPEL	6MT3	B*18:01	2.08	4.16
5IM7	B*58:01	QASQEVKNW	5IND	B*58:01	1.54	2.30
5INC	B*58:01	QATQEVANW	5IM7	B*58:01	1.87	2.71
5IND	B*58:01	QASQDVKNW	5IM7	B*58:01	1.30	1.88
5MEO	A*02:01	ILGKFLHRL	5MEP	A*02:01	1.14	1.62
5MEP	A*02:01	ILGKFLHWL	4I4W	A*02:01	0.92	2.30
5MEQ	A*02:01	ILAKFLHTL	5MER	A*02:01	0.75	2.19
5MER	A*02:01	ILAKFLHEL	5MEQ	A*02:01	1.40	1.81
5N1Y	A*02:01	MVWGPDPLYV	5C0F	A*02:01	1.63	2.18
5N6B	A*02:01	LLWNGPMAV	6SS8	A*02:01	1.13	2.10
5NMH	A*02:01	SLYNTIATL	2V2W	A*02:01	0.75	1.32
5NMK	A*02:01	SLFNTIAVL	2V2X	A*02:01	1.37	3.08
5SWQ	A*02:01	CVNGSCFTV	3MRJ	A*02:01	1.00	1.33
5T6W	B*57:01	SSTRGISQLW	5T6X	B*57:01	1.01	1.45
5T6X	B*57:01	TSTTSVASSW	5T6W	B*57:01	0.95	1.25
5T6Z	B*57:01	TSTLQEQIGW	5T70	B*57:01	3.58	5.11
5T70	B*57:01	TSNLQEQIGW	5T6Z	B*57:01	3.17	4.66
5TXS	B*15:01	AQDIYRASY	1XR8	B*15:01	1.63	3.43
5U98	B*57:01	VTTDIQVKV	5VUF	B*57:01	2.30	3.73
5V5L	B*58:01	TSTLQEQIGW	5T70	B*57:01	2.98	4.17
5VGD	C*05:01	SAEPVPLQL	6ULI	C*08:02	1.95	3.01
5VGE	C*07:02	RYRPGTVAL	5W6A	C*06:02	2.36	3.11
5VUD	B*57:01	LSSPVTKSW	3VH8	B*57:01	0.64	0.96
5VUE	B*57:01	LTVQVARVW	5VUF	B*57:01	0.91	1.70
5VUF	B*57:01	LTVQVARVY	5VUE	B*57:01	1.11	1.61
5VVP	B*57:03	LSSPVTKSW	6V2Q	B*57:03	1.81	2.87
5VWD	B*57:03	LTVQVARVW	5VWF	B*57:03	2.45	2.96
5VWF	B*57:03	LTVQVARVY	5VWD	B*57:03	0.94	2.08
5VWH	B*58:01	LSSPVTKSW	5IM7	B*58:01	2.21	2.82
5VWJ	B*58:01	LTVQVARVW	5VWH	B*58:01	0.76	1.36
5VZ5	B*15:01	AQDIYRASYY	2AXG	B*35:01	2.24	4.21
5W69	C*06:02	ARFNDLRFV	5W6A	C*06:02	0.60	2.28
5W6A	C*06:02	ARTELYRSL	5W69	C*06:02	1.16	2.98
5WJN	A*11:01	GTSGSPIINR	5WKF	A*11:01	1.18	1.78
5WKF	A*11:01	GTSGSPIVNR	5WJN	A*11:01	0.98	1.28
5WMN	B*07:02	SPIVPSFDM	4U1K	B*07:02	0.55	1.04
5WMO	B*07:02	RPPIFIRRL	4U1K	B*07:02	2.46	5.13
5WMP	B*07:02	TPRVTGGGAM	6UJ8	B*07:02	2.47	3.01
5WMQ	B*08:01	ELRSRYWAI	1M05	B*08:01	1.97	2.57
5WMR	B*08:01	QIKVRVDMV	4QRQ	B*08:01	1.58	2.26
5WSH	A*02:01	GVWIRTPTA	5E00	A*02:01	1.76	2.57
5XOS	B*35:01	IPLTEEAEL	6BJ8	B*35:01	1.25	1.56
5XS3	C*06:02	VRSRRCLRL	5W6A	C*06:02	0.80	2.31
6AM5	A*02:01	SMLGIGIVPV	3MRR	A*02:01	1.21	1.53
6AMU	A*02:01	MMWDRGLGMM	5N1Y	A*02:01	2.64	4.85
6AT9	A*01:01	AQDIYRASYY	1QVO	A*11:01	2.66	3.50
6BJ8	B*35:01	VPLTEDAEL	5XOS	B*35:01	0.96	1.24
6D29	B*57:01	TSMSFVPRPW	5T6X	B*57:01	2.81	3.73
6D2T	B*57:01	LALLTGVRW	5VUE	B*57:01	1.86	2.57
6EWC	A*02:01	RLSSPLHFV	1I1Y	A*02:01	1.63	3.05
6G3J	A*02:01	MTSAIGILPV	4JFP	A*02:01	0.95	1.30
6J1W	A*30:01	AIFQSSMTK	6J29	A*30:03	1.69	2.02
6JOZ	A*11:01	ATIGTAMYK	1Q94	A*11:01	1.65	1.88
6MT3	B*18:01	FEDLRVLSF	4QRR	B*35:01	1.62	2.31
6MT4	B*37:01	FEDLRVSSF	6MT6	B*37:01	1.18	1.35
6MT5	B*37:01	FEDLRLLSF	6MT6	B*37:01	1.37	1.60
6MT6	B*37:01	FEDLRVLSF	6MT5	B*37:01	0.93	1.30
6MTL	B*44:05	FEDLRVLSF	1SYV	B*44:05	1.89	3.70

			Template		RMSD	
**PDB**	**MHC-I**	**Epitope**	**PDB**	**MHC-I**	**Backbone**	**Interface**
6O4Z	A*02:01	KLVVVAVGV	6O4Y	A*02:01	1.31	1.64
6O51	A*02:01	YLVVVGAVGV	6O53	A*02:01	1.44	1.91
6O53	A*02:01	KLVVVGAVGV	6O51	A*02:01	1.56	1.89
6O9B	A*03:01	TTAPSLSGK	6O9C	A*03:01	0.83	1.30
6O9C	A*03:01	TTAPFLSGK	6O9B	A*03:01	1.01	1.40
6OPD	A*02:01	ILNAMIVKI	6PTB	A*02:01	0.79	1.71
6PBH	A*68:01	DATALVR	5GSD	A*11:01	2.66	3.50
6PTB	A*02:01	ILNAMIAKI	6OPD	A*02:01	1.62	2.21
6PTE	A*02:01	ILNAMITKI	6PTB	A*02:01	1.14	1.85
6PYW	B*27:05	LRNQSVFNF	3LV3	B*27:05	0.72	1.47
6Q3K	A*02:01	NLVPMVATV	3GSR	A*02:01	0.85	1.52
6R2L	A*02:01	SLSKILDTV	5MEQ	A*02:01	0.98	2.03
6SS7	A*02:01	LLWAGPMAV	5N6B	A*02:01	1.33	2.06
6SS8	A*02:01	LLWNGPIAV	5N6B	A*02:01	1.23	2.12
6SS9	A*02:01	LLWNGPMHV	6SSA	A*02:01	0.94	1.64
6SSA	A*02:01	LLWNGPMQV	6SS9	A*02:01	0.93	1.76
6TRN	A*02:01	AVYDGREHTV	1I4F	A*02:01	0.86	1.23
6UJ7	B*07:02	SPNGTIQNIL	6UJ8	B*07:02	1.43	1.70
6UJ8	B*07:02	SPNGTIRNIL	6UJ7	B*07:02	0.83	1.50
6UJO	A*02:06	KQWLVWLFL	6UJQ	A*02:06	1.36	2.10
6UJQ	A*02:06	KQWLVWLLL	6UJO	A*02:06	1.65	2.64
6V2O	B*57:01	ASLNLPAVSW	5T6X	B*57:01	4.55	4.11
6V2P	B*57:03	ASLNLPAVSW	5T6X	B*57:01	3.63	4.40
6V2Q	B*57:03	LSSPVTKSF	5VVP	B*57:03	1.83	2.78
6VMC	A*02:01	ILDQVPFSV	1TVH	A*02:01	1.22	1.89
6VR1	A*02:01	HMTEVVRRC	6VR5	A*02:01	1.21	2.20
6VR5	A*02:01	HMTEVVRHC	6VR1	A*02:01	0.83	1.81
6XQA	A*24:02	TYQWVLKNL	7JYW	A*24:02	0.72	0.96
6Z9V	A*02:01	IIGWMWIPV	6Z9W	A*02:01	1.73	4.35
6Z9W	A*02:01	LLGWVFAQV	6Z9V	A*02:01	1.50	2.62
7CIQ	B*27:05	RRFSRSPIRR	4G8I	B*27:05	2.39	4.06
7EJL	A*24:02	QYIKWPWYI	7EJN	A*24:02	1.01	2.46
7EJM	A*24:02	TYIKWPWWV	7EJL	A*24:02	1.09	2.31
7EJN	A*24:02	MYVKWPWYV	7EJL	A*24:02	1.15	2.27
7EU2	A*02:01	KIADYNYKL	5HHP	A*02:01	0.99	2.33
7F4W	A*24:02	NYNYLYRLF	7JYW	A*24:02	1.40	2.91
7JYU	A*24:02	IYFSPIRVTF	4F7M	A*24:02	1.40	2.38
7JYV	A*24:02	YFSPIRVTF	2BCK	A*24:02	1.08	2.26
7JYW	A*24:02	TYQWIIRNW	6XQA	A*24:02	0.79	1.41
7KGO	A*02:01	ILLNKHIDA	3FT4	A*02:01	1.02	1.92
7KGP	A*02:01	GMSRIGMEV	6R2L	A*02:01	1.35	2.50
7KGQ	A*02:01	LLLDRLNQL	5MER	A*02:01	1.00	1.86
7KGR	A*02:01	LQLPQGTTL	3MRD	A*02:01	1.81	3.02
7KGS	A*02:01	ALNTPKDHI	6EWC	A*02:01	1.59	3.25
7L1B	A*03:01	AHHGGWTTK	7L1C	A*03:01	0.72	2.45
7L1C	A*03:01	ALHGGWTTK	7L1B	A*03:01	0.85	1.73
7LGD	B*07:02	SPRWYFYYL	7LGT	B*07:02	2.15	5.28
7LGT	B*07:02	SPKLHFYYL	7LGD	B*07:02	2.01	4.46
7N1A	A*02:01	YLQPRTFLL	5EU6	A*02:01	1.32	2.61
7N1E	A*02:01	RLQSLQTYV	7KGT	A*02:01	0.88	2.14

Supplemental Table 2: FlexPepDock refinement benchmark results summary, including PDB structures used for the benchmark and the PDB structures used as templates.

Peptide	Sequence	Protein	Gene	NCBI Reference Sequence	Start	-	End
P1	LAGKNLTHI	sodium/glucose cotransporter 2	Slc5a2	NP_573517.1	303	-	311
P2	SQNKNAKAM	solute carrier family 22 member 2 isoform 1	Slc22a2	NP_038695.1	296	-	304
P3	FSYKKMTIM	inositol oxygenase	Miox	NP_064361.2	66	-	74
P4	SSSKPVEPL	podocin	Nphs2	NP_569723.1	372	-	380
P5	KAMKIIKHI	solute carrier family 22 member 2 isoform 1	Slc22a2	NP_038695.1	302	-	310
P6	YILKLPLPL	cadherin-16 isoform 1 precursor	Cdh16	NP_031689.1	42	-	50
P7	GSPKQHEVV	solute carrier family 23 member 1	Slc23a1	NP_035527.3	8	-	16
P8	AAPDHKVVV	cadherin-16 isoform 1 precursor	Cdh16	NP_031689.1	531	-	539
P9	FSLQVKPEI	hepatitis A virus cellular receptor 1 homolog isoform b precursor	Havcr1	NP_001160104.1	124	-	132
P10	MQLPSKVVL	kelch domain-containing protein 7A	Klhdc7a	NP_775603.2	17	-	25
P11	SVPTHKSSL	solute carrier organic anion transporter family member 4C1	Slco4c1	NP_766246.1	255	-	263
P12	MADITKSYL	N-acetyltransferase 8	Nat8	NP_075944.1	95	-	103
P13	MMKFNKFSL	inositol oxygenase	Miox	NP_064361.2	203	-	211

Supplemental Table 3: List of peptide candidates for *in vitro* and *in vivo* screening.

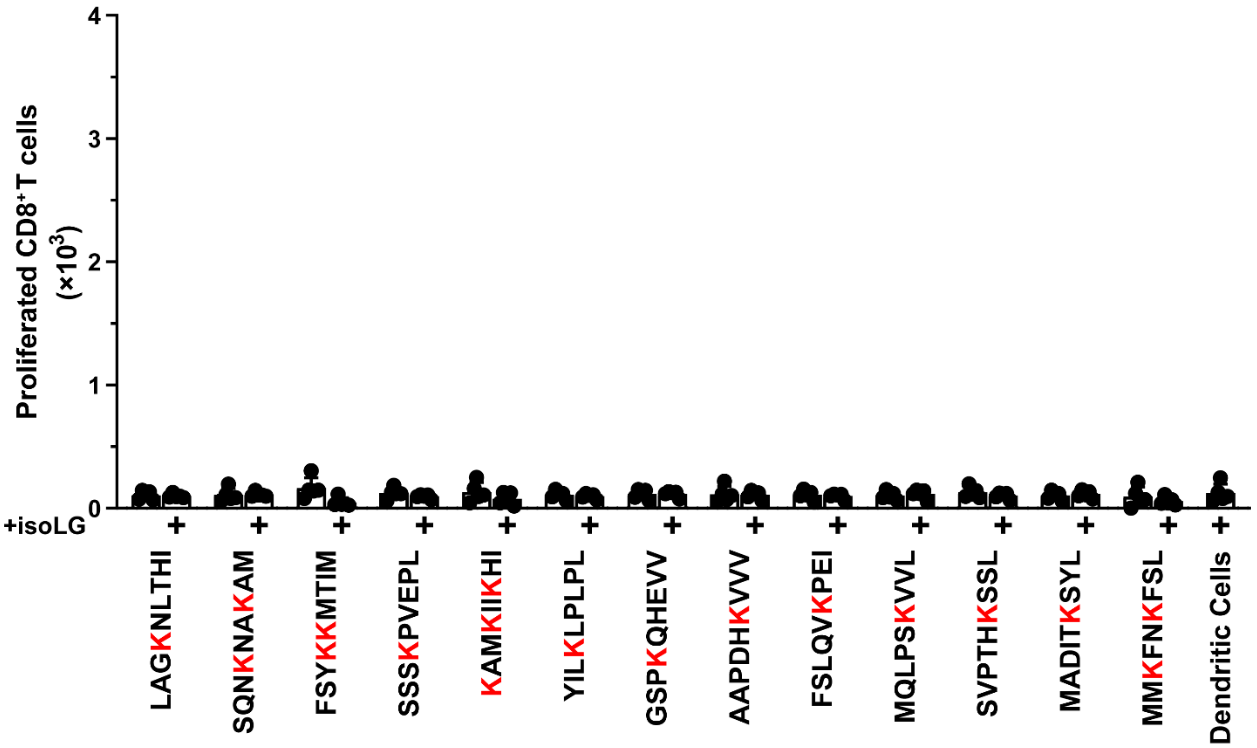

Supplemental Figure 3: T cells isolated from sham treated mice do not proliferate when exposed to isoLG-adducted peptides. n=5, mean ± S.D., student's t-test.

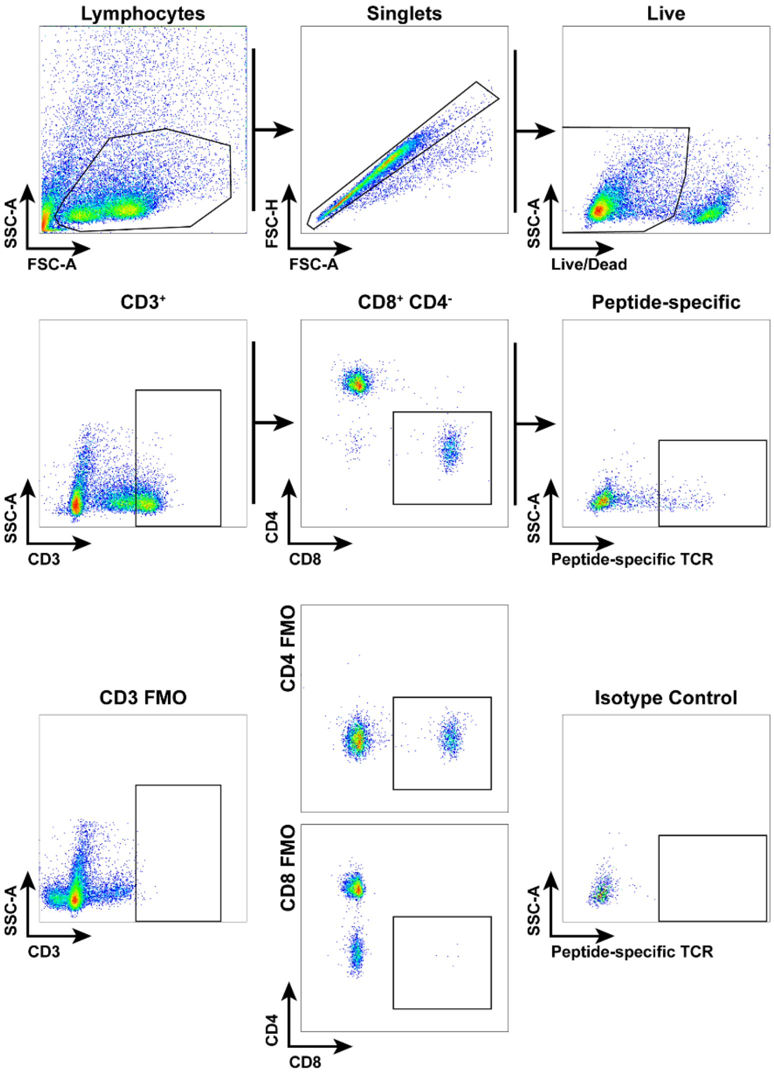

Supplemental Figure 4: Gating strategy and FMOs used for identifying peptide-specific CD8$^+$ T cells in peripheral tissues.

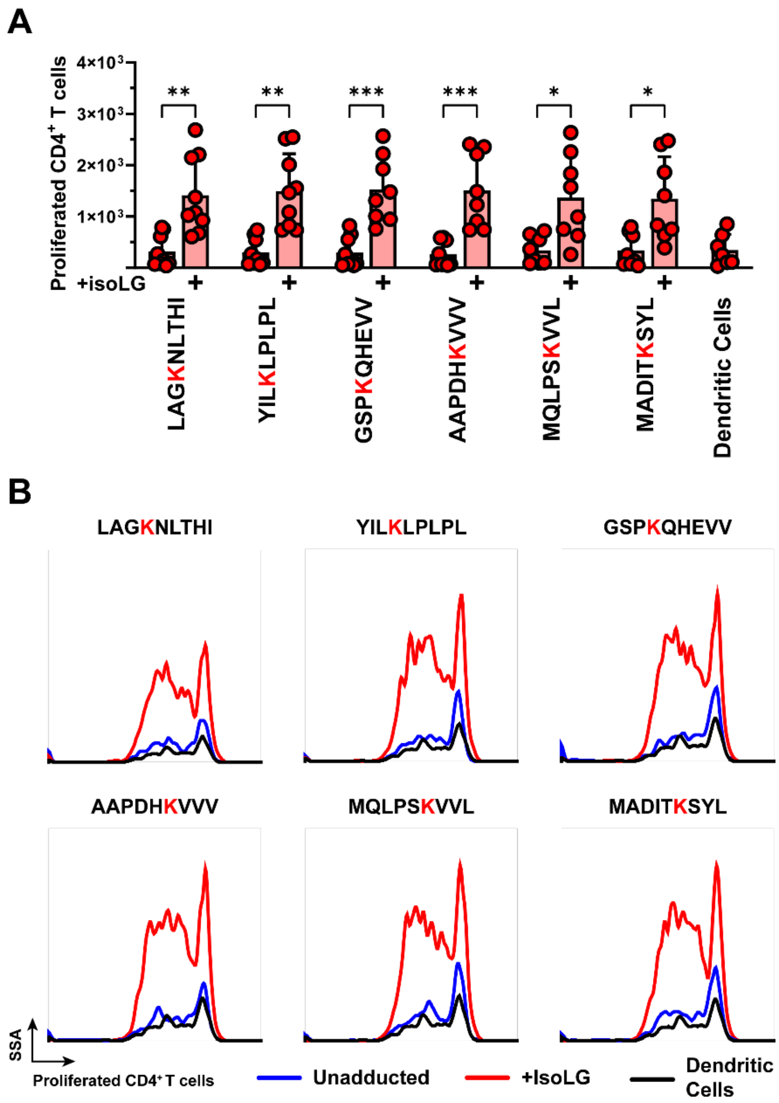

Supplemental Figure 5: IsoLG-adducted peptides induce CD4⁺ T cell proliferation *in vitro*. (A) Six candidate IsoLG-adducted peptides induce proliferation of $CD4^+$ T cells isolated from the bone marrow of hypertensive mice (n=7-9, mean ± S.D., *p<0.05, **p<0.01, ***p<0.001, student's t-test). (B) Representative CFSE dye-dilution curves illustrating $CD4^+$ T cell proliferation after five days of incubation with dendritic cells and IsoLG-adducted peptides.

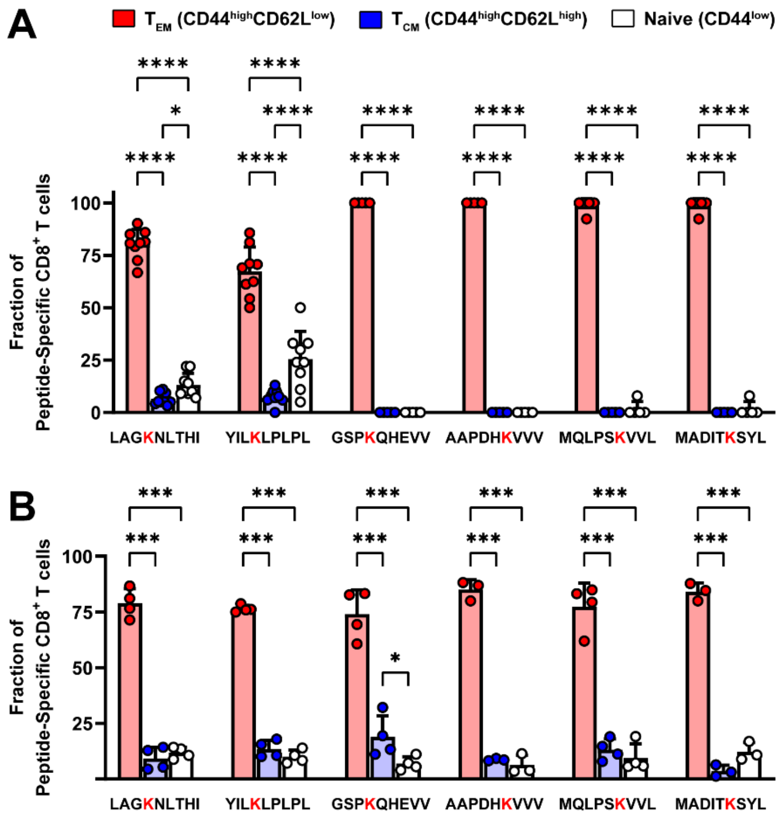

Supplemental Figure 6: Peptide-specific memory CD8^{+} T cells are present in the aorta and kidneys of normotensive mice. (A) The majority of peptide-specific CD8^{+} T cells in the aorta (A) and the kidney (B) are effector memory T cells in normotensive mice (n=3-9, mean ± S.D., **p*<0.05, ***p*<0.01, ****p*<0.001, 2-way ANOVA and Holm-Sidak post-hoc).

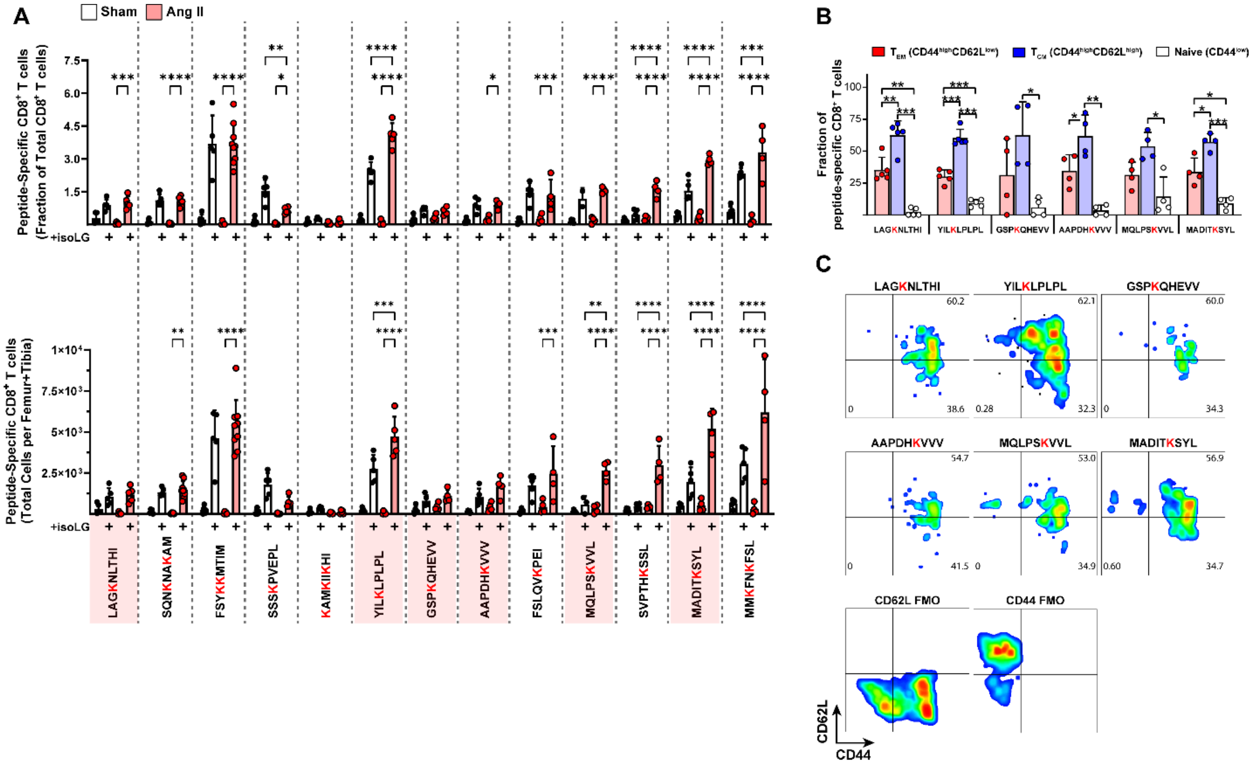

Supplemental Figure 7: CD8+ T cells recognizing IsoLG-adducted peptides are enriched in the bone marrow for some, but not all, peptides of interest. (A) The fraction and number of peptide-specific CD8$^+$ T cells are significantly enriched in the bone marrow for only two of the six peptides of interest, highlighted in red (n=3-9, 2-way ANOVA and Holm-Sidak post-hoc). (B) IsoLG-adducted peptide-specific CD8$^+$ T cells in the bone marrow are predominantly a mixture of effector and central memory cell (n=4-5, mean ± S.D., (**p*<0.05, ***p*<0.01, ****p*<0.001, 2-way ANOVA and Holm-Sidak post-hoc).

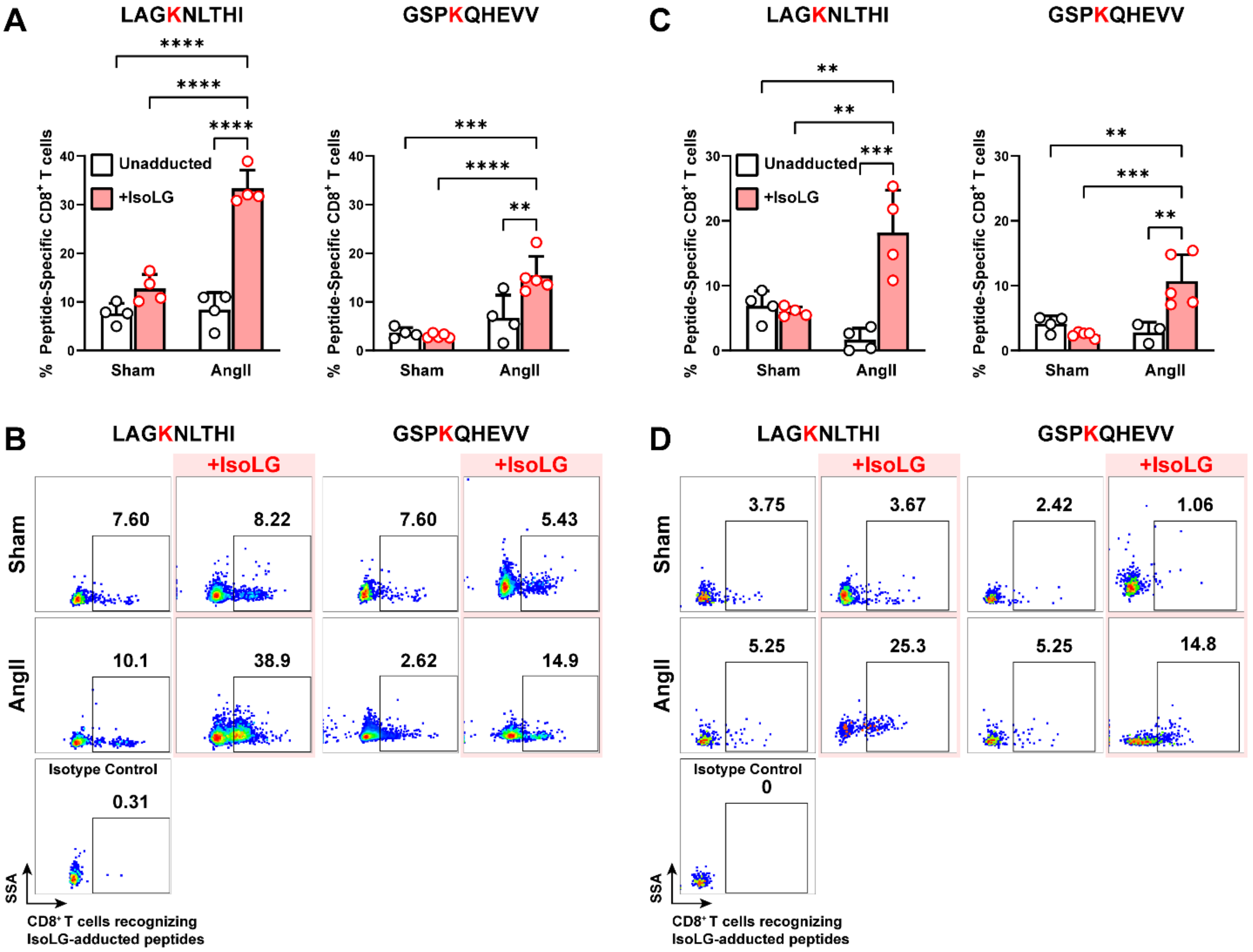

Supplemental Figure 8: IsoLG-adducted peptide-specific CD8$^+$ T cells are increased in the aortas and kidneys of hypertensive mice following adoptive transfer and low-dose angiotensin II infusion. The fraction of peptide-specific CD8$^+$ T cells is increased in the aortas (A-B) and kidneys (C-D) of mice following adoptive transfer of IsoLG-adducted peptides and infusion of low-dose angiotensin II for two weeks. Adoptive transfer alone or low-dose angiotensin II alone is insufficient to cause an enrichment of peptide-specific T cells in these tissues. (n=4, mean ± S.D., ****p*<0.01, *****p*<0.001, ******p*<0.0001, 2-way ANOVA and Holm-Sidak post-hoc).

		Number of alleles selected for screening		
Population Name	Size	**HLA-A**	**HLA-B**	**HLA-C**
USA NMDP European Caucasian	1,242,890	5	8	2
USA NMDP African American pop 2	416,581	4	5	1
USA NMDP Chinese	99,672	4	5	1
	**Total unique alleles**	**5**	**10**	**3**

Supplemental Table 4: Population sizes and HLA alleles screened for IsoLG-presentation. Three of the largest USA NMDP populations available in the HLA allele frequency database were queried, and alleles selected based on frequency of occurrence in that population (>5% phenotype frequency). Duplicate alleles across populations are counted only once in the total selected for screening.

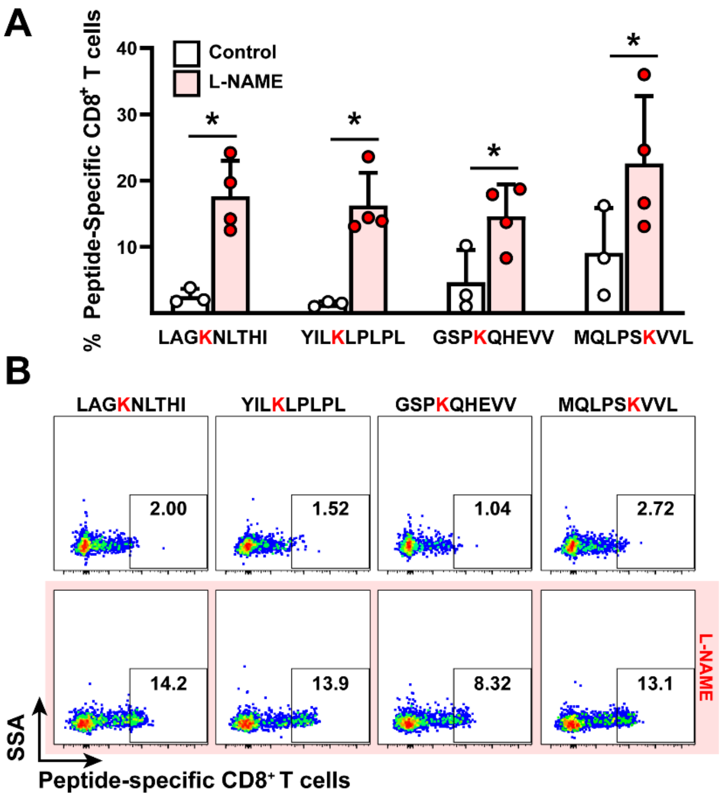

Supplemental Figure 9: CD8^{+} T cells recognizing IsoLG adducts increase in prevalence after induction of hypertension with L-NAME. (A) There is a significant increase in the fraction of CD8^{+} T cells recognizing IsoLG-adducted peptides in the aortas of mice treated with L-NAME compared to untreated controls (n=4, mean ± S.D., *p<0.01, student's t-test). (B) Representative flow plots demonstrating the increase in IsoLG-adducted peptide-specific T cells.

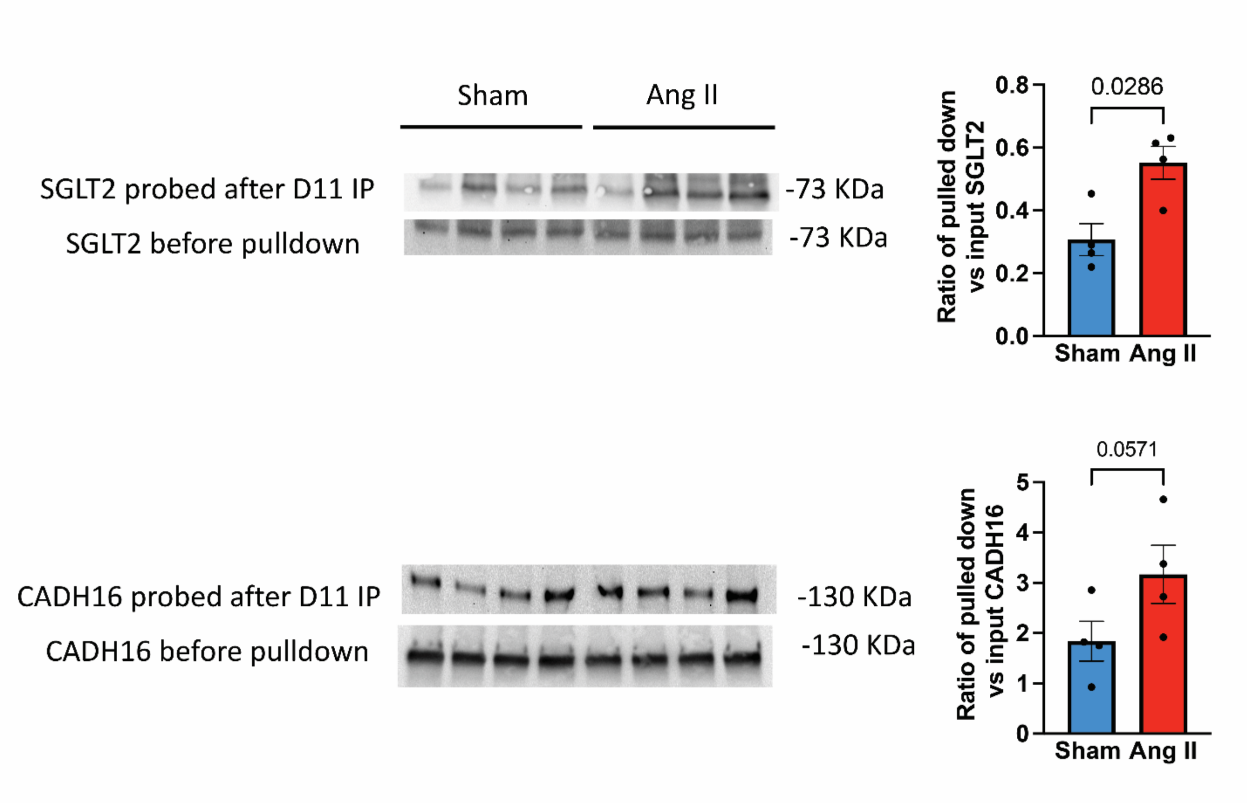

Supplemental Figure 10: The relative abundance of IsoLG-adducted proteins from which immunogenic peptides are derived is significantly increased in kidney homogenates following treatment with angiotensin II. n=4, mean ± S.E., student's t-test.

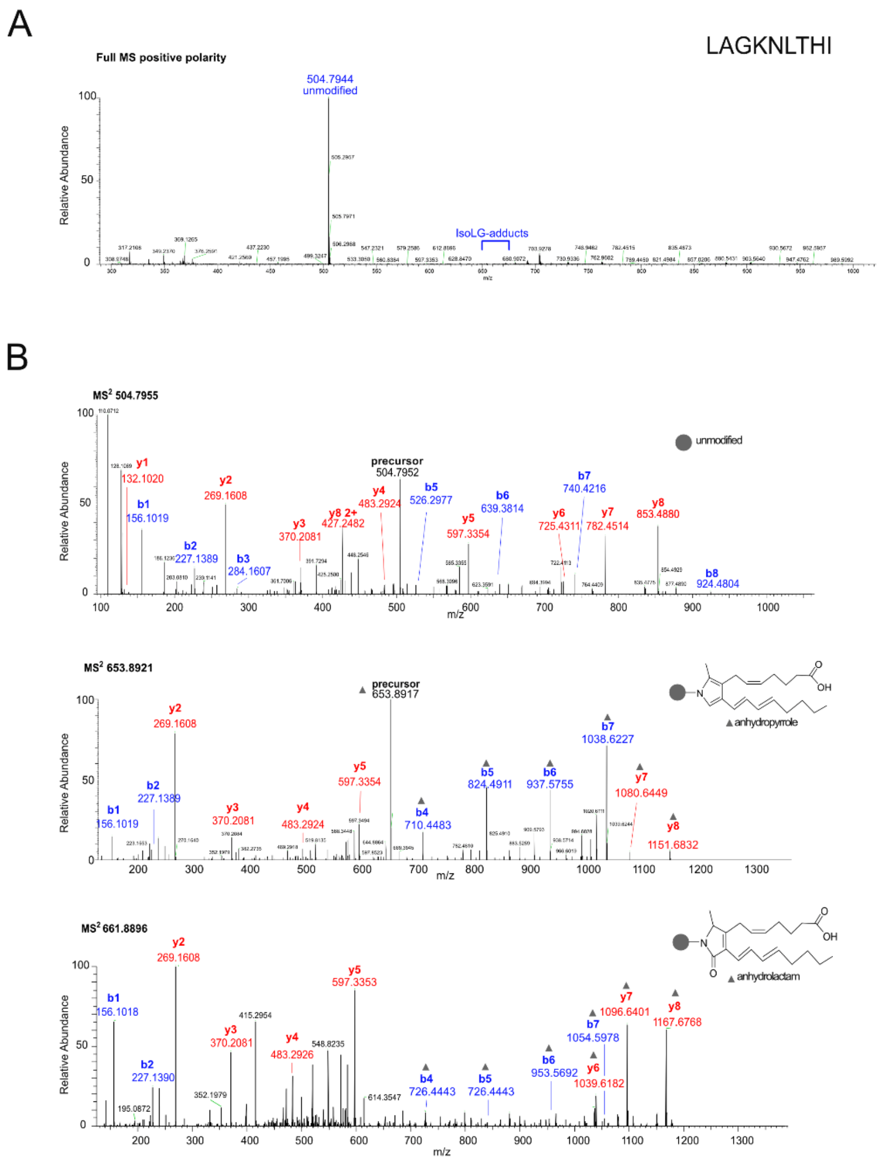

Supplemental Figure 11: Mass spectrometric analysis of the isolevuglandin-E15 (IsoLG) reaction products of the peptide Acetyl-LAGKNLTHI. A) Positive polarity MS of the reaction mixture. IsoLG adducted peptide ions are detectable, but at signal intensities that are two to three orders of magnitude lower than the un-modified peptide. B) MS2 HCD fragmentation spectra of the unmodified peptide, the anhydropyrrole-IsoLG and the anhydrolactam-IsoLG adducted peptide. Series of b- and y- ions are labeled in blue or red, respectively. Fragment ions that carry the IsoLG modification are marked with a solid grey triangle.

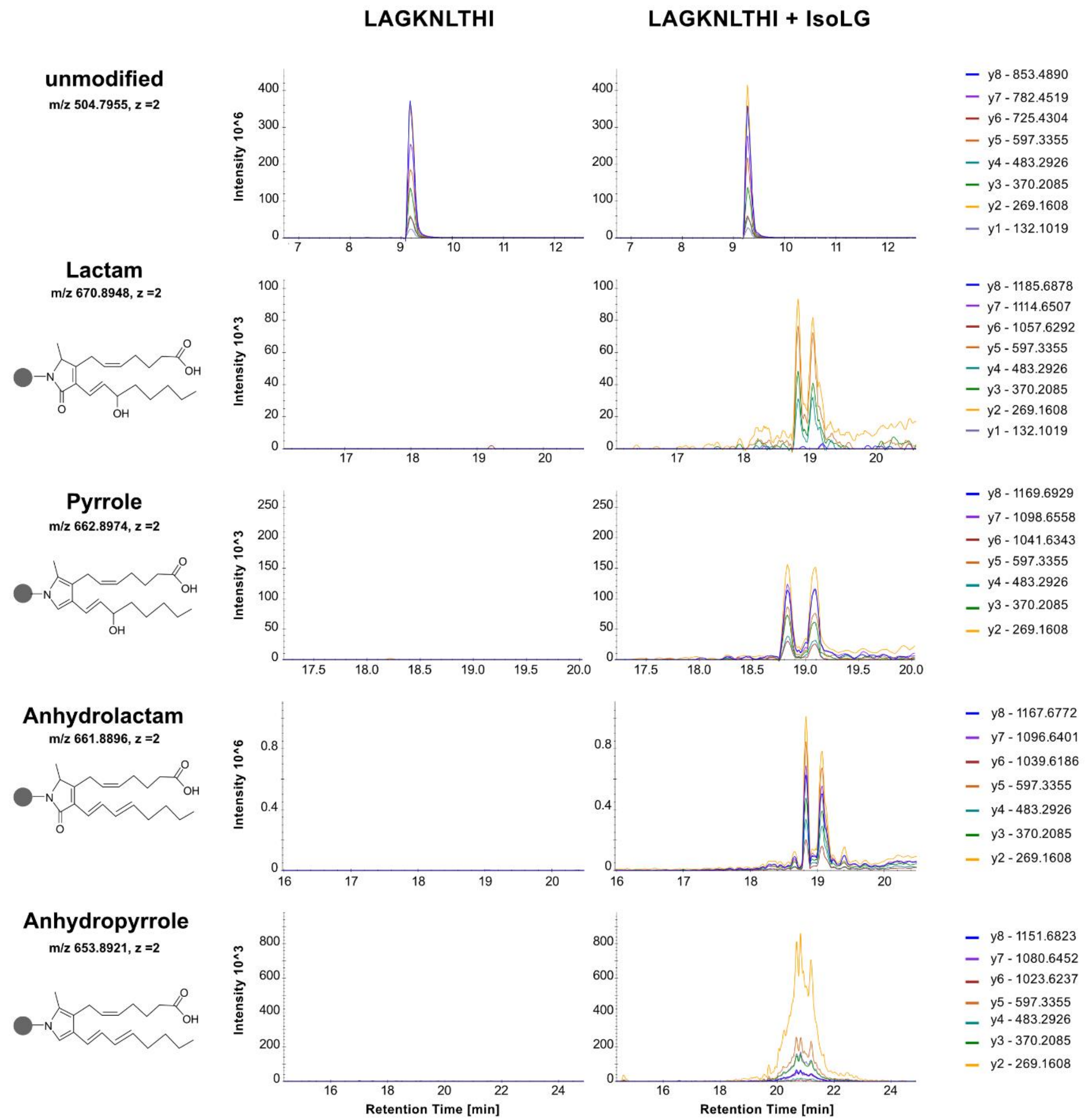

Supplemental Figure 12: Targeted mass spectrometric orbitrap parallel reaction monitoring (PRM) analysis of the immunogenic peptide Acetyl-LAGKNLTHI without and after treatment with isolevuglandin-E15. Extracted ion chromatograms are shown for each PRM transition as singly charged y ions. The formation of isobaric IsoLG adduct diastereomers leads to the observation of multiple complex chromatographic peaks for the pyrrole, anhydrolactam, and anhydropyrrole products, simplified structures of which are indicated. The grey circle represents the peptide; its lysine ε-nitrogen atom is included in the adduct structure.

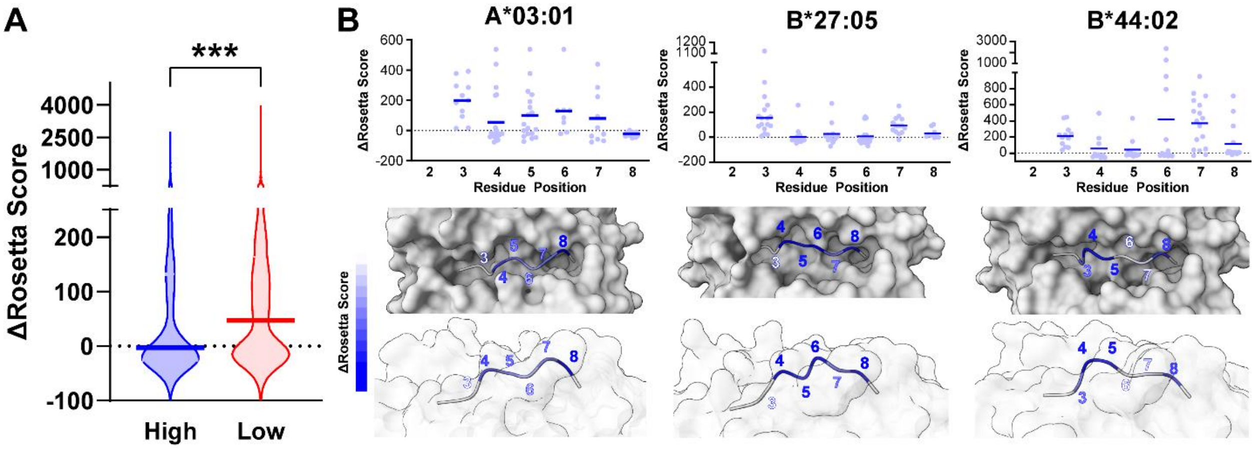

Supplemental Figure 13: Rosetta predicts more favorable energy changes following isoLG adduction for peptides complexed with "high presenting" HLA alleles. (A) Rosetta energy changes are significantly lower (more favorable) following isoLG adduction for peptides complexed with the five high presenting HLA-A and HLA-B alleles compared to the lower-presenting variants (n=472 for High presenters, n=975 for Low presenters, mean value indicated by solid bars, ***p<0.0001, Mann-Whitney test). (B) Per-residue energy changes following isoLG adduction for peptides bound to high presenters with available crystal structures (top, mean value indicated by solid bars). Residues with more favorable changes are shown in darker blue, and generally correspond to regions of the epitope that jut up and out of the binding cleft (bottom).
